# Supplementary material for: The respiratory microbiome is linked to the severity of RSV infections and the persistence of symptoms in children
Source: Cell Rep Med. 2024 Dec 5;5(12):101836. doi: 10.1016/j.xcrm.2024.101836 (PMC11722103; doi:10.1016/j.xcrm.2024.101836)
Supplement: Document S1. Figures S1–S4 and Tables S1–S4 [file mmc1.pdf]

Cell Reports Medicine, Volume *5*

Supplemental information

The respiratory microbiome is linked to the severity of RSV infections and the persistence of symptoms in children

Maartje Kristensen, Wouter A.A. de Steenhuijsen Piters, Joanne Wildenbeest, Marlies A. van Houten, Roy P. Zuurbier, Raiza Hasrat, Kayleigh Arp, Mei Ling J.N. Chu, Marie Billard, Terho Heikkinen, Steve Cunningham, Matthew Snape, Simon B. Drysdale, Ryan S. Thwaites, Federico Martinon-Torres, Andrew J. Pollard, Peter J.M. Openshaw, Jeroen Aerssen, Justyna Binkowska, Louis Bont, Debby Bogaert, and REspiratory Syncytial virus Consortium in EUrope (RESCEU) investigators

Cell Reports Medicine, Volume *5*

Supplemental information

The respiratory microbiome is linked to the severity of RSV infections and the persistence of symptoms in children

Maartje Kristensen, Wouter A.A. de Steenhuijsen Piters, Joanne Wildenbeest, Marlies A. van Houten, Roy P. Zuurbier, Raiza Hasrat, Kayleigh Arp, Mei Ling J.N. Chu, Marie Billard, Terho Heikkinen, Steve Cunningham, Matthew Snape, Simon B. Drysdale, Ryan S. Thwaites, Federico Martinon-Torres, Andrew J. Pollard, Peter J.M. Openshaw, Jeroen Aerssen, Justyna Binkowska, Louis Bont, Debby Bogaert, and REspiratory Syncytial virus Consortium in EUrope (RESCEU) investigators

Supplemental information

Variable	Overall, N = 773[1]	No RSV infection in the first year of life, N = 576[1]	RSV infection in the first year of life, N = 197[1]	p-value[2]
Year of birth				0.004[a]
2017	56/773 (7.2%)	41/576 (7.1%)	15/197 (7.6%)	
2018	336/773 (43%)	270/576 (47%)	66/197 (34%)	
2019	381/773 (49%)	265/576 (46%)	116/197 (59%)	
Season of birth				0.459[a]
Autumn	186/773 (24%)	146/576 (25%)	40/197 (20%)	
Spring	198/773 (26%)	148/576 (26%)	50/197 (25%)	
Summer	225/773 (29%)	165/576 (29%)	60/197 (30%)	
Winter	164/773 (21%)	117/576 (20%)	47/197 (24%)	
Gestational age (weeks)				0.942[a]
37+0-37+6	67/760 (8.8%)	52/568 (9.2%)	15/192 (7.8%)	
38+0-38+6	98/760 (13%)	74/568 (13%)	24/192 (12%)	
39+0-39+6	240/760 (32%)	178/568 (31%)	62/192 (32%)	
>=40+0	355/760 (47%)	264/568 (46%)	91/192 (47%)	
Vaginal delivery	212/773 (27%)	156/576 (27%)	56/197 (28%)	0.715[a]
Birth weigth	3,490 (3,158, 3,780)	3,470 (3,122, 3,789)	3,516 (3,286, 3,748)	0.084[b]
Female sex	380/773 (49%)	290/576 (50%)	90/197 (46%)	0.259[a]
Apgar score (1 minute)	9.00 (9.00, 9.00)	9.00 (9.00, 9.00)	9.00 (9.00, 9.00)	0.147[b]
Apgar score (5 minutes)	10.00 (9.00, 10.00)	10.00 (9.00, 10.00)	10.00 (9.00, 10.00)	0.872[b]
Apgar score (10 minutes)	10.00 (9.00, 10.00)	10.00 (9.00, 10.00)	10.00 (9.00, 10.00)	0.310[b]
Postnatal infection	18/770 (2.3%)	15/574 (2.6%)	3/196 (1.5%)	0.584[c]
Postnatal antibiotic treatment	13/18 (72%)	12/15 (80%)	1/3 (33%)	0.172[c]
Asphyxia	3/769 (0.4%)	2/573 (0.3%)	1/196 (0.5%)	>0.999[c]
Hospitalisation	78/773 (10%)	54/576 (9.4%)	24/197 (12%)	0.259[a]
Hospital length of stay	2.00 (2.00, 3.00)	2.00 (2.00, 3.00)	2.00 (2.00, 3.00)	0.699[b]
Any respiratory support	8/766 (1.0%)	5/571 (0.9%)	3/195 (1.5%)	0.427[c]
Mother planning to breastfeed	680/771 (88%)	506/574 (88%)	174/197 (88%)	0.949[a]
Intention to register to daycare	368/764 (48%)	259/568 (46%)	109/196 (56%)	0.016[a]
Maternal smoking during pregnancy	40/771 (5.2%)	34/574 (5.9%)	6/197 (3.0%)	0.116[a]
Household smoking	101/770 (13%)	81/573 (14%)	20/197 (10%)	0.153[a]
Pets inside the home	319/771 (41%)	253/574 (44%)	66/197 (34%)	0.009[a]
Any family member with history of asthma	206/768 (27%)	146/572 (26%)	60/196 (31%)	0.165[a]
Number of RTI episodes	1.00 (0.00, 3.00)	1.00 (0.00, 2.00)	3.00 (2.00, 4.00)	<0.001[b]
Age at first RSV infection	170 (108, 260)	N/A	170 (108, 260)	
Medically attended RSV infection	100/185 (54%)	N/A	100/185 (54%)	
Severity of first RSV infection				
mild	173/190 (91%)	N/A	173/190 (91%)	
moderate	17/190 (8.9%)	N/A	17/190 (8.9%)	

Table S1. Baseline characteristics of early-life samples, stratified by whether or not the child would develop an RSV infection in the first year of life.

[1]n/N (%); Median (IQR)

[2]To assess statistical significance between groups (subjects who will develop an RSV infection over the first year of life and those who do not) we used a [a]Pearson's Chi-squared test; [b]Wilcoxon rank sum test or a [c]Fisher's exact test.

Variable	Healthy controls, N = 52[1]	RSV, N = 374[1]	p-value[2]
Study			<0.001[a]
Birth cohort	0/52 (0%)	117/374 (31%)	
Case-control	52/52 (100%)	257/374 (69%)	
Age (days)	222 (112, 297)	94 (48, 200)	<0.001[b]
Female sex	15/52 (29%)	174/373 (47%)	0.016[a]
Year (visit)			<0.001[c]
2017	0/52 (0%)	22/254 (8.7%)	
2018	11/52 (21%)	106/254 (42%)	
2019	41/52 (79%)	100/254 (39%)	
2020	0/52 (0%)	26/254 (10%)	
Season (visit)			<0.001[c]
Autumn	21/52 (40%)	163/254 (64%)	
Spring	11/52 (21%)	1/254 (0.4%)	
Summer	19/52 (37%)	0/254 (0%)	
Winter	1/52 (1.9%)	90/254 (35%)	
Site			0.063[c]
Spain	6/52 (12%)	27/347 (7.8%)	
The Netherlands	25/52 (48%)	181/347 (52%)	
UK (London)	3/52 (5.8%)	3/347 (0.9%)	
UK (Oxford)	18/52 (35%)	136/347 (39%)	
Duration onset of symptoms to sampling (days)	N/A	4.00 (3.00, 5.00)	
ReSViNET score	N/A	6.0 (4.0, 10.0)	
Fever (>38.5°C)	0/52 (0%)	317/374 (85%)	<0.001[a]
Any medical attendance	0/52 (0%)	301/370 (81%)	<0.001[a]
Hospitalisation	0/52 (0%)	192/367 (52%)	<0.001[a]
PICU admission	0/52 (0%)	61/241 (25%)	<0.001[a]
Any comorbidities	29/51 (57%)	40/257 (16%)	<0.001[a]

Table S2. Baseline characteristics of healthy controls compared to RSV cases.

[1]n/N (%); Median (IQR)

[2]To assess statistical significance between groups (healthy controls compared to RSV cases) we used a [a]Pearson's Chi-squared test; [b]Wilcoxon rank sum test or a [c]Fisher's exact test.

Variable	Mild RSV, N = 218[1]	Moderate RSV, N = 106[1]	Severe RSV, N = 47[1]	p-value[2]
Study				<0.001[a]
Birth cohort	106/218 (49%)	10/106 (9.4%)	1/47 (2.1%)	
Case-control	112/218 (51%)	96/106 (91%)	46/47 (98%)	
Age (days)	158 (62, 247)	87 (48, 184)	48 (28, 96)	<0.001[b]
Female sex	106/217 (49%)	49/106 (46%)	18/47 (38%)	0.418[a]
Year (visit)				0.738[c]
2017	8/112 (7.1%)	9/95 (9.5%)	5/44 (11%)	
2018	45/112 (40%)	40/95 (42%)	20/44 (45%)	
2019	50/112 (45%)	34/95 (36%)	15/44 (34%)	
2020	9/112 (8.0%)	12/95 (13%)	4/44 (9.1%)	
Season (visit)				0.031[c]
Autumn	82/112 (73%)	55/95 (58%)	24/44 (55%)	
Spring	0/112 (0%)	1/95 (1.1%)	0/44 (0%)	
Winter	30/112 (27%)	39/95 (41%)	20/44 (45%)	
Site				0.007[c]
Spain	9/194 (4.6%)	15/104 (14%)	2/46 (4.3%)	
The Netherlands	112/194 (58%)	42/104 (40%)	27/46 (59%)	
UK (London)	2/194 (1.0%)	0/104 (0%)	1/46 (2.2%)	
UK (Oxford)	71/194 (37%)	47/104 (45%)	16/46 (35%)	
Duration onset of symptoms to sampling (days)	3.00 (3.00, 5.00)	4.00 (3.00, 5.00)	4.00 (3.00, 5.00)	0.072[b]
ReSViNET score	4.0 (3.0, 6.0)	10.0 (8.2, 11.8)	16.0 (14.5, 17.0)	<0.001[b]
Fever (>38.5°C)	200/218 (92%)	80/106 (75%)	36/47 (77%)	<0.001[a]
Any medical attendance	152/214 (71%)	101/106 (95%)	47/47 (100%)	<0.001[a]
Hospitalisation	53/214 (25%)	93/105 (89%)	45/45 (100%)	<0.001[a]
PICU admission	4/101 (4.0%)	23/93 (25%)	34/46 (74%)	<0.001[a]
Any comorbidities	8/112 (7.1%)	19/96 (20%)	13/46 (28%)	0.002[a]

Table S3. Baseline characteristics of RSV cases, stratified by severity.

[1]n/N (%); Median (IQR)

[2]To assess statistical significance between groups (mild, moderate and severe RSV) we used a [a]Pearson's Chi-squared test; [b]Kruskal-Wallis rank sum test or a [c]Fisher's exact test.

Study	Group	Mean	SD	Min	p25	median	p75	Max
Birth cohort	Baseline	31,141	15,553	5,008	19,579	30,777	41,453	76,940
	RSV	35,680	7,913	12,298	30,640	36,063	39,904	63,236
	RSV (conv)	37,500	7,948	18,534	32,579	36,136	40,296	63,245
Case-control	Healthy controls	27,218	5,120	12,091	24,793	26,542	30,150	40,964
	RSV	27,207	8,100	5,383	22,060	26,952	30,957	57,902
	RSV (conv)	27,509	7,150	13,426	22,106	26,621	31,917	46,772
Overall	Overall	30,586	12,620	5,008	22,580	29,890	37,878	76,940

Table S4. Sequencing depth stratified by study and study group. Number of reads (after quality control/decontamination) stratified by study and study groups. ‘Overall’ represents the sequencing depth across the total dataset. Mean, standard deviation (SD), minimum (Min; p0), maximum (Max; p100), Median (p50) and 25th (p25) and 75th (p75) percentiles are shown.

Figure S1

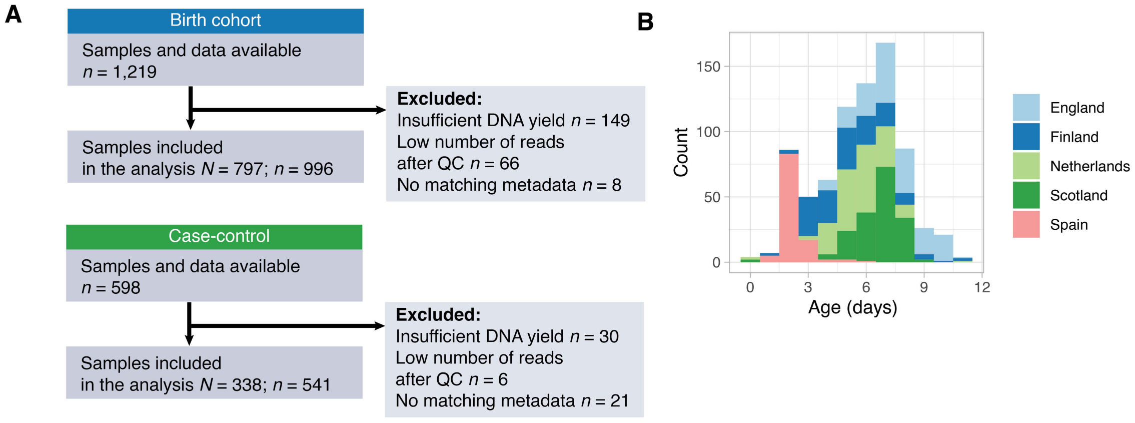

Figure S1. Sample inclusion and age at baseline sample collection. (A) Flow chart indicating the number of participants (N) and samples (n) at each step during laboratory processing and data preparation. (B) Histogram showing the age at sample collection of baseline samples per study site.

Figure S2

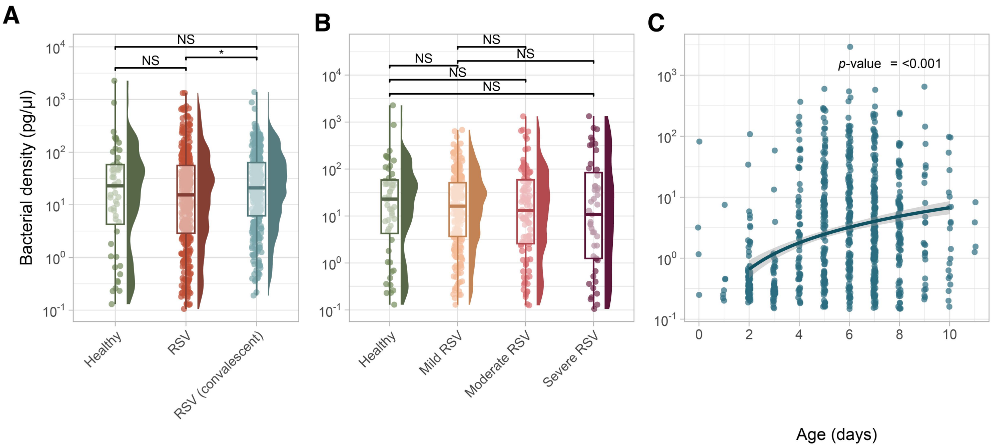

Figure S2. Bacterial density between groups and across age. (A) Bacterial density in healthy controls, during and after RSV infection and (B) in healthy controls (*n* = 52), mild (RESViNet-score 0-7; *n* = 218), moderate (8-13; *n* = 106) and severe RSV infection (14-20; *n* = 47). Statistical significance was assessed using linear mixed effects models with log_{10}-transformed bacterial density as outcome, age, sex and health status (healthy controls, RSV or RSV convalescent; A) or RSV severity (healthy, mild, moderate and severe RSV; B) as fixed effects and study site as random effect. Subject ID was additionally included for comparisons between RSV and RSV convalescence. Box plots represent the 25th and 75th percentiles (lower and upper boundaries of boxes, respectively), the median (middle horizontal line), and measurements that fall within 1.5 times the interquartile range (IQR; distance between 25th and 75th percentiles; whiskers). (C) Bacterial density for baseline samples over age. Statistical significance was assessed using a linear mixed effects model, including log_{10}-transformed bacterial density as outcome, the natural log of age, season of birth, sex and presence of siblings as fixed effects and study site as random effect. Asterisks denote statistical significance (NS, not significant [$p > 0.05$]; *, $p \leq 0.05$; **, $p \leq 0.01$; ***, $p \leq 0.001$).

Figure S3

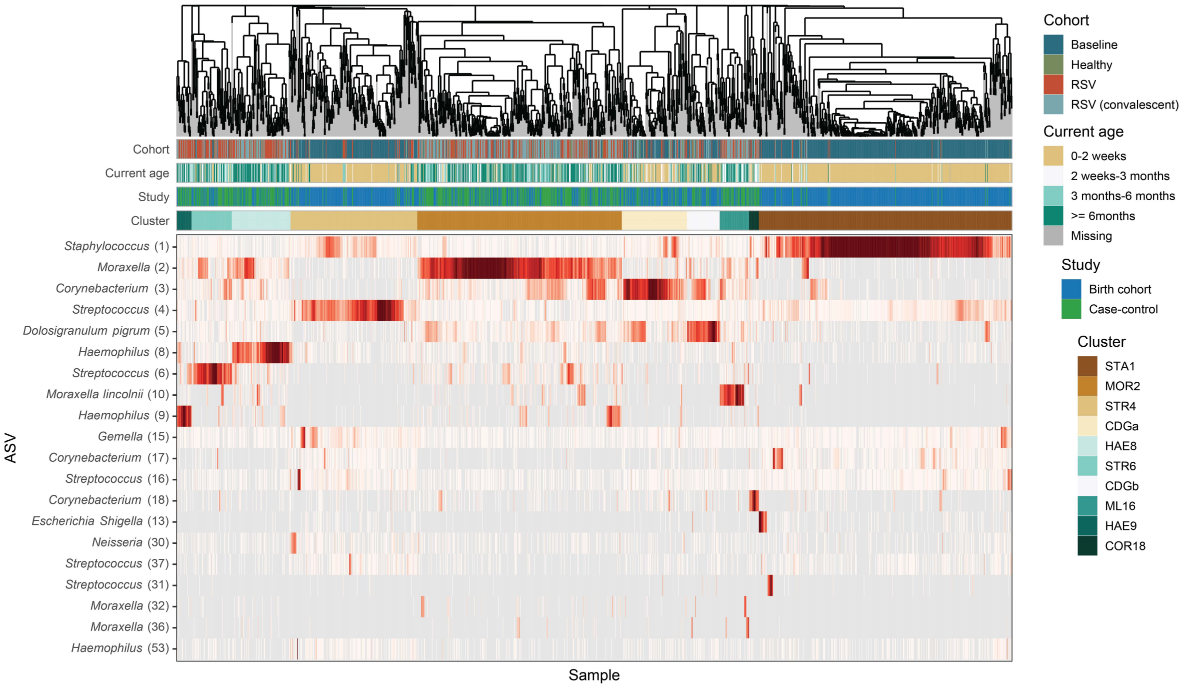

Figure S3. Dendrogram visualizing a complete linkage hierarchical clustering of samples based on the Bray-Curtis dissimilarity matrix. The length of the branches of the tree structure corresponds with the similarities between samples (*n* = 1,537). Adjacent to the branch ends information on 1) cohort [baseline, healthy controls, RSV and RSV convalescent], 2) current age, 3) study [birth cohort or case-control] and 4) cluster membership is depicted. Based on Calinski- Harabasz and Silhouette-indices we found an optimum of 10 clusters. These included clusters characterized by *Staphylococcus* (1) (STA1; *n* = 466), *Moraxella* (2) (MOR2; *n* = 376), *Streptococcus* (4) (STR4; *n* = 233), *Corynebacterium* (3)/*Dolosigranulum pigrum* (5) (CDGa and CDGb; *n* = 180), *Haemophilus* (8) (HAE8; *n* = 108), *Streptococcus* (6) (STR6; *n* = 74), *Moraxella lincolnii* (16) (ML16; *n* = 54), *Haemophilus* (9) (HAE9; *n* = 28) or *Corynebacterium* (18) (COR18; *n* = 18). A heatmap shows the relative abundance of the 20 highest-ranked ASVs based on mean relative abundance across all samples. Repeated samples from individuals were included in this clustering analysis to optimize cluster identification and increase comparability across time points.

Figure S4

Group(s)	Model	Dependent variable	Independent variables	Figure
HC RSVc / RSV HC	lmer	α-diversity	health status, age, sex, read count*, study site	2A
RSV RSVc	lmer	α-diversity	health status, age, sex, read count*, time RSV/RSVc, study site, subject ID	2A
HC RSVc / RSV HC	lmer	density†	health status, age, sex, study site	S2A
RSV RSVc	lmer	density†	health status, age, sex, study site, subject ID	S2A
HC RSVc / RSV HC	PERMANOVA	β-diversity	health status, age, sex, study site	2B
RSV RSVc	PERMANOVA	β-diversity	health status, age, sex, time RSV/RSVc, study site	
RSV RSVc	PERMANOVA	β-diversity	health status, age, subject ID	
HC RSVc / RSV HC	lmer	health status	cluster, age, sex, study site	2D
HC RSVc / RSV HC	MaAsLin2	ASV/genus†	health status, age, sex, study site	2E-F
HC mild / HC mod / HC sev	lmer	α-diversity	severity, age, sex, read count*, study site	3A
HC mild / HC mod / HC sev	lmer	density†	severity, age, sex, study site	S2B
HC mild / HC mod / HC sev	PERMANOVA	β-diversity	severity, age, sex, study site	3B
RSVc mild / RSVc mod / RSVc sev	lmer	stability	severity, age, sex, time RSV/RSVc, study site	3C
HC mild / HC mod / HC sev	lmer	severity	cluster, age, sex, study site	3D
HC mild / HC mod / HC sev	MaAsLin2	ASV/genus†	severity, age, sex, study site	3E-F
HC mild / HC mod / HC sev	MaAsLin2	ASV/genus†	severity, age, sex, time RSV/RSVc, abx, study site (within RSVc)	
<symptoms>	MaAsLin2	ASV/genus†	symptom, age, sex, time RSV/RSVc, study site (within RSVc)	4A-B
baseline	lmer	α-diversity	age†, sex, read count*, birth season, siblings, study site	5A
baseline	lmer	density†	age†, sex, birth season, siblings, study site	S2C
baseline	PERMANOVA	β-diversity	age (cat), study site (+/- Spain)	5B
baseline	PERMANOVA	β-diversity	study site, age (cat)	
baseline	PERMANOVA	β-diversity	birth season, age (cat), study site	
baseline	PERMANOVA	β-diversity	birth mode, age (cat), study site	
baseline	PERMANOVA	β-diversity	feeding, age (cat), study site	
baseline	PERMANOVA	β-diversity	sex, age (cat), study site	
baseline	PERMANOVA	β-diversity	siblings, age (cat), study site	
no RSV any RSV	lmer	RSV y/n	cluster, age, sex, birth season, siblings, study site	
no RSV any RSV	PERMANOVA	β-diversity	RSV y/n, age, sex, birth season, siblings, study site	
non-MA RSV MA RSV	lmer	MA RSV y/n	cluster, age, sex, age RSV, birth season, siblings, study site	5D
non-MA RSV MA RSV	PERMANOVA	β-diversity	MA RSV y/n, age, sex, age RSV, birth season, siblings, study site	
no RSV any RSV	rf	RSV y/n	ASV1, ASV2 ... ASV39, age RSV	
non-MA RSV MA RSV	rf	MA RSV y/n	ASV1, ASV2 ... ASV39, age RSV	5E-G

Variable of interest | Fixed effects | Optional effect | Random effects/blocked permutations

Figure S4. Overview of statistical models. Overview of all (multivariable) models included in either main text, (supplementary) figures or both. For each model, the type of model, dependent variables and independent variables (stratified by fixed effects or random effects) are indicated. Models were ran with and without 'optional effects'. Linear or logistic mixed effects models (lmerTest::lmer or lme4::glmer, respectively) were applied for continuous or dichotomous dependent variables, respectively, including study site and/or subject ID as random effect throughout. Blocked permutations were applied in PERMANOVA-tests to account for study site and/or subject ID. * scaled; † log$_{10}$- or natural log-transformed; HC, healthy controls; RSVc, RSV convalescence; mod, moderate RSV infection; sev, severe RSV infection; age (cat), age categories; MA, medically attended; rf, random forest.
