# Supplementary material for: The respiratory microbiome is linked to the severity of RSV infections and the persistence of symptoms in children
Source: Cell Rep Med. 2024 Dec 5;5(12):101836. doi: 10.1016/j.xcrm.2024.101836 (PMC11722103; doi:10.1016/j.xcrm.2024.101836)
Supplement: Document S2. Article plus supplemental information [file mmc2.pdf]

Cell Reports
Medicine

Article

The respiratory microbiome is linked to the severity of RSV infections and the persistence of symptoms in children

Graphical abstract

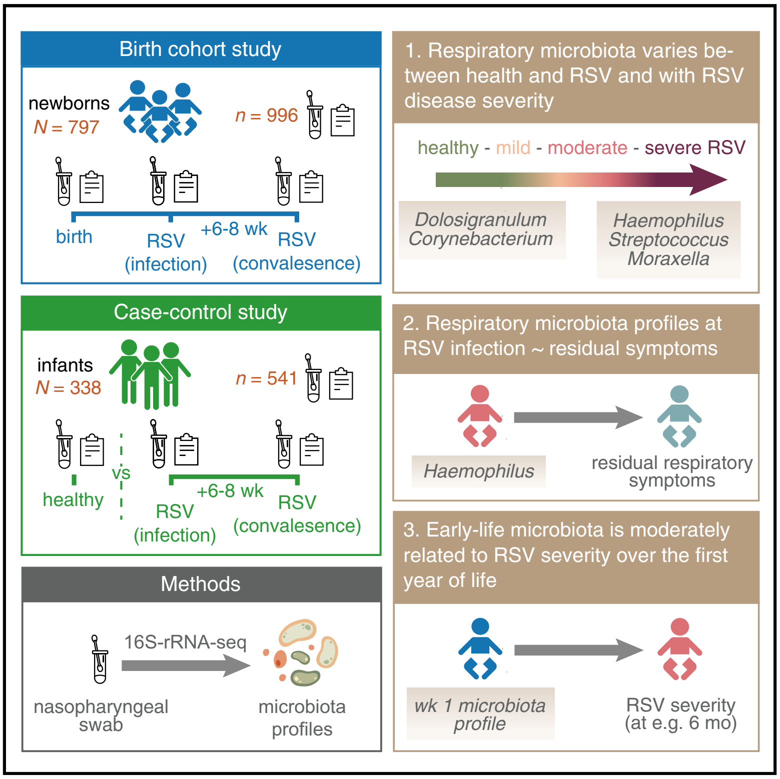

Highlights

- The neonatal respiratory microbiota is only modestly associated with RSV severity
- Respiratory microbiota during RSV infection differs between RSV cases and controls
- *Haemophilus*, *Streptococcus*, and *Moraxella* are linked to severe disease
- *Dolosigranulum* and *Corynebacterium* are associated with milder disease and health

Authors

Maartje Kristensen,
Wouter A.A. de Steenhuijsen Piters,
Joanne Wildenbeest, ..., Louis Bont,
Debby Bogaert, REspiratory Syncytial virus Consortium in EUrope (RESCEU) investigators

Correspondence

d.bogaert@ed.ac.uk

In brief

In this study, Kristensen et al. investigate the link between the respiratory microbiome and RSV severity. Although the neonatal respiratory microbiota is only modestly associated with RSV severity during the first year of life, the respiratory microbiota during RSV infection is strongly associated with disease severity and persistence of symptoms.

Kristensen et al., 2024, Cell Reports Medicine *5*, 101836
December 17, 2024 © 2024 The Authors. Published by Elsevier Inc.
https://doi.org/10.1016/j.xcrm.2024.101836

CellPress

Cell Reports Medicine

CellPress OPEN ACCESS

Article

The respiratory microbiome is linked to the severity of RSV infections and the persistence of symptoms in children

Maartje Kristensen,[1,2,13] Wouter A.A. de Steenhuijsen Piters,[1,2,13] Joanne Wildenbeest,[1] Marlies A. van Houten,[3] Roy P. Zuurbier,[3] Raiza Hasrat,[1,2] Kayleigh Arp,[1,2] Mei Ling J.N. Chu,[1,2] Marie Billard,[1] Terho Heikkinen,[4] Steve Cunningham,[5] Matthew Snape,[6] Simon B. Drysdale,[6,7] Ryan S. Thwaites,[8] Federico Martinon-Torres,[9,10,11] Andrew J. Pollard,[6] Peter J.M. Openshaw,[8] Jeroen Aerssen,[12] Justyna Binkowska,[5] Louis Bont,[1] Debby Bogaert,[1,5,14,*] and REspiratory Syncytial virus Consortium in EUrope (RESCEU) investigators

[1]Department of Paediatric Immunology and Infectious Diseases, Wilhelmina Children's Hospital/University Medical Center Utrecht, Utrecht, the Netherlands

[2]Centre for Infectious Disease Control, National Institute for Public Health and the Environment, Bilthoven, the Netherlands

[3]Department of Paediatric Diseases, Spaarne Gasthuis, Haarlem and Hoofddorp, the Netherlands

[4]Department of Pediatrics, University of Turku and Turku University Hospital, Turku, Finland

[5]Centre for Inflammation Research, Queen's Medical Research Institute, University of Edinburgh, Edinburgh, UK

[6]Oxford Vaccine Group, Department of Paediatrics, University of Oxford, and the NIHR Oxford Biomedical Research Centre, Oxford, UK

[7]Centre for Neonatal and Paediatric Infection, St George's, University of London, London, UK

[8]National Heart & Lung Institute, Imperial College, London, UK

[9]Genetics, Vaccines, and Infectious Diseases Research Group (GENvip, www.genvip.eu), Instituto de Investigación Sanitaria de Santiago, Universidad de Santiago de Compostela, Galicia, Spain

[10]Centro de Investigación Biomédica en Red de Enfermedades Respiratorias (CIBERES), Madrid, Spain

[11]Translational Pediatrics and Infectious Diseases, Pediatrics Department, Hospital Clínico Universitario de Santiago, Santiago de Compostela, Spain

[12]Janssen Pharmaceutica, Beerse, Belgium

[13]These authors contributed equally

[14]Lead contact

*Correspondence: d.bogaert@ed.ac.uk

https://doi.org/10.1016/j.xcrm.2024.101836

SUMMARY

Respiratory syncytial virus (RSV) is the leading cause of infant respiratory infections and hospitalizations. To investigate the relationship between the respiratory microbiome and RSV infection, we sequence nasopharyngeal samples from a birth cohort and a pediatric case-control study (Respiratory Syncytial virus Consortium in Europe [RESCEU]). 1,537 samples are collected shortly after birth ("baseline"), during RSV infection and convalescence, and from healthy controls. We find a modest association between baseline microbiota and the severity of consecutive RSV infections. The respiratory microbiota during infection clearly differs between infants with RSV and controls. *Haemophilus*, *Streptococcus*, and *Moraxella* abundance are associated with severe disease and persistence of symptoms, whereas stepwise increasing abundance of *Dolosigranulum* and *Corynebacterium* is associated with milder disease and health. We conclude that the neonatal respiratory microbiota is only modestly associated with RSV severity during the first year of life. However, the respiratory microbiota at the time of infection is strongly associated with disease severity and residual symptoms.

INTRODUCTION

Respiratory syncytial virus (RSV) is the most common cause of lower respiratory tract infections and hospitalizations in infants.[1] Known risk factors for severe infections are preterm birth, congenital heart disease, and bronchopulmonary dysplasia.[2–4] However, even in term-born healthy infants, RSV infection can lead to severe illness, requiring admission to the pediatric intensive care unit, and may lead to long-term health consequences, such as persistent wheeze.[5] The fact that RSV severity can vary between a common cold and severe bronchiolitis with respiratory failure indicates other host factors or environmental factors may modulate disease severity.

Several studies have found a link between the early-life respiratory microbial community composition and subsequent risk of (caretaker-reported) respiratory tract infections.[6–9] Though mechanisms are not fully understood, these findings may be explained by immunomodulation of microbiota or immunological

© 2024 The Authors. Published by Elsevier Inc.
This is an open access article under the CC BY license (http://creativecommons.org/licenses/by/4.0/).

imprinting of early-life microbial events. As of yet, it is still unknown whether the early-life microbiome is also specifically linked to the severity of RSV infections.

Previous studies in infants, however, have indicated that nasopharyngeal microbiota are associated with RSV infection occurrence,[10] severity,[11–13] and the risk of developing recurrent wheezing and childhood asthma.[14,15] Specifically, multiple studies have consistently reported associations between the abundance of potential pathogens *Haemophilus* spp. and *S. pneumoniae*[13,16] and RSV disease severity. Recent evidence suggests that the combination of microbial community composition, inflammatory parameters, and clinical severity during RSV infection is associated with the risk of childhood asthma.[17] Most evidence of microbial shifts during RSV and its long-term consequences has so far been based on studies in children who were hospitalized with RSV infections, thus focusing on moderately to severely ill children. However, the majority of children who contract RSV experience mild disease.[18]

In this current study, we have the unique opportunity to investigate the variation in microbial community composition (1) preceding RSV infection, e.g., using materials obtained in the first 10 days of life, (2) during acute infection, and (3) during the convalescent phase, across the full range of RSV disease severity, from very mild not medically attended infections to moderate and severe RSV infections. We hypothesize that (1) early-life microbiota is associated with the occurrence and/or severity of consecutive RSV disease, (2) the occurrence and severity of RSV infection are associated with specific respiratory microbial profiles, and (3) microbial profiles are linked to (persistent) respiratory symptoms during convalescence.

RESULTS

Cohort description

We were able to use samples from two pediatric cohorts executed by the large European Union-funded Respiratory Syncytial virus Consortium in Europe (RESCEU). In total, we analyzed 1,537 nasopharyngeal samples collected from 1,135 infants across two RESCEU studies executed in five countries, including a case-control and a birth cohort study. Study inclusion has been previously published.[18,19] For the case-control study, we included infants with RSV infections during the first year of life who presented with symptoms to family care practices or emergency departments. Additionally, we included a group of healthy infants.[20] For the birth cohort study, we followed infants throughout the first year of life in an actively followed-up nested cohort and tested them for RSV if they experienced respiratory symptoms during the RSV season.[21] These studies were executed across five countries between 2017 and 2020. We included 797 infants (996 samples) from the birth cohort, and 257 RSV cases (489 samples) and 52 healthy controls (52 samples) from the case-control study (Figures S1A and 1). A higher percentage of females were included in the birth cohort compared to the case-control study (49% vs. 44%, respectively; Pearson's chi-squared test; p value = 0.025), and the median age at first RSV infection was lower in the case-control compared to the birth cohort study (median [interquartile range (IQR)] 105 [49–209] (recorded for N = 190 children) vs. 170 [109–260] days, respectively; Wilcoxon rank-sum test; p value < 0.001). Clinical baseline characteristics of infants can be found in Tables S1–S3.

Baseline samples were collected at a median age of 6 days (range 0–11 days), though samples from Spain were collected earlier, at a median age of 2 days (range 1–6 days; Figure S1B). RSV cases from both the case-control study and the birth cohort were generally younger compared to healthy controls (median [IQR] 94 [48–200] compared with 222 [112–297] days, respectively; Wilcoxon rank-sum test; p < 0.001), were more often male (47% vs. 29%, Pearson's chi-squared test; p value = 0.016), and RSV samples were mainly collected in autumn and winter (64% and 35%, respectively; Fisher's exact test, p < 0.001). RSV cases identified within the birth cohort study were mostly mild (90.6% of total RSV cases), whereas RSV cases in the case-control study were, as expected, more evenly distributed in relation to severity (mild, moderate, and severe in 43.6%, 37.4%, and 17.9%, respectively). Convalescent samples were collected 6–8 weeks after infection.

From the 1,557 samples collected, 1,135 samples were available for downstream analysis after sequencing. Samples were sequenced at an average sequence depth of 30,652 reads (interquartile range 22,718–37,940) (Table S4), after removal of contaminating reads.

Bacterial microbiota during RSV infection and at convalescence

Microbial diversity in healthy controls and in infants with RSV infection

First, we investigated microbiota profiles during RSV infection (n = 374) and during RSV convalescence (n = 338; collected at a median [IQR] 52 [48–58] days after RSV) and compared these to profiles of healthy controls (n = 52). No significant difference in alpha-diversity was detected between samples from healthy controls and those of infants during RSV infections (Figure 2A). Alpha-diversity (Shannon) was, however, lower at convalescence when compared to healthy controls (linear mixed-effects model adjusted for age, gender, sequencing depth, and study site [random effect]; $\beta = -0.212$, p value = 0.005). For the latter analyses, the results remained similar when including the time between RSV and convalescence as a covariate. When comparing the number of observed amplicon sequence variants (ASVs) (richness) between groups, richness was lower at convalescence when compared to during RSV infection ($\beta = -0.073$, p value = 4.5×10^{-3}). Inversely, $\log_{10}$-transformed bacterial density was higher at convalescence compared to RSV infection (Figure S2A; $\beta = 0.138$, p value = 0.022).

Using principal coordinate analysis (PCoA), we found distinct differences in overall microbial community composition between control samples, RSV samples, and convalescent samples. We estimated the association between health status and the overall microbial community, while adjusting for age at sample collection and gender (restricting permutations within study site), showing that acute RSV and convalescent samples significantly differed from healthy controls (PERMANOVA; R^2 = 1.0%, p value < 0.001 and R^2 = 1.2%, p value < 0.001, respectively). In addition, we found that samples collected at convalescence differed from acute RSV (R^2 = 1.2%, p value < 0.001; Figure 2B),

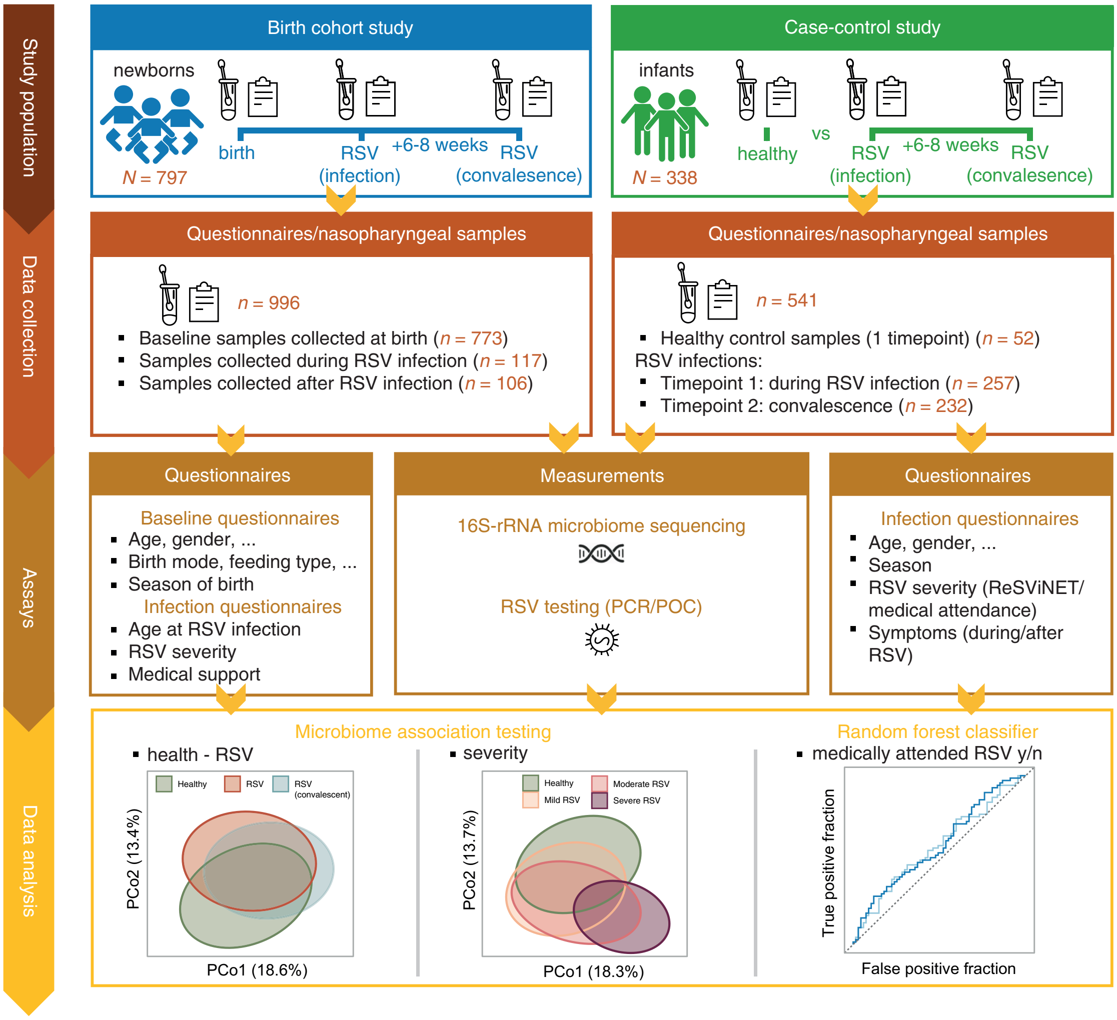

Figure 1. Study overview

Experimental setup to assess (1) the relationship between microbiota profiles preceding RSV infection (early-life) on later-life RSV occurrence and severity, (2) microbiota profiles during/after RSV infection compared to healthy controls, and (3) severity-associated changes in microbiota profiles. Samples were included from two studies, conducted across five European study sites. From all newborns/infants, questionnaires were collected on demographics, RSV risk factors, and measures of RSV severity. In addition, (longitudinal) nasopharyngeal samples were collected, resulting in matched samples collected before (birth cohort only), during, and after RSV infection (birth cohort and case-control study). All samples were subjected to 16S-rRNA-sequencing to characterize bacterial microbiota profiles. Linear/logistic mixed-effects regression models were employed, allowing us to adjust for relevant covariates and account for study site (random effect) (Methods). *N*, number of individuals; *n*, number of samples.

which remained significant when including the time between infection and convalescence in our model (R^2 = 1.1%, *p* value < 0.001) or when restricting permutations within subject (R^2 = 1.1%, *p* value = 0.001). In these models, we observed an effect of age on the overall microbial community composition (adjusted for health status, gender, and restricting permutations by study site) of R^2 = 1.7% (*p* value < 0.001).

Clustering of healthy controls, RSV, and convalescent samples

To define clusters or profile types within our samples, we applied unsupervised, complete linkage hierarchical clustering to the complete dataset, binning samples with similar microbiota profiles together, resulting in microbiota clusters (Figure S3). Samples clustered predominantly in a *Moraxella* (2)-dominated cluster (MOR2; *n* = 346, 45%; case-control study), followed by a *Haemophilus* (8) (HAE8; *n* = 104, 14%), a *Corynebacterium* (3) plus *Dolosigranulum* (4) (CDGa/b; *n* = 90, 12%), and a *Streptococcus* (6)-dominated cluster (STR6; *n* = 66, 8.6%; Figure 2C). The ASV annotated as *Streptococcus* (6) demonstrated 100% sequence similarity with *S. (pseudo)pneumoniae* isolates only (BLASTn), suggesting that this ASV represents pneumococcus, a known potential pathogen. Sensitivity analyses, using only a single sample per subject (*n* = 1,111), demonstrated high consistency with the clustering based on the full dataset, with 96.2% of samples assigned to the same clusters.

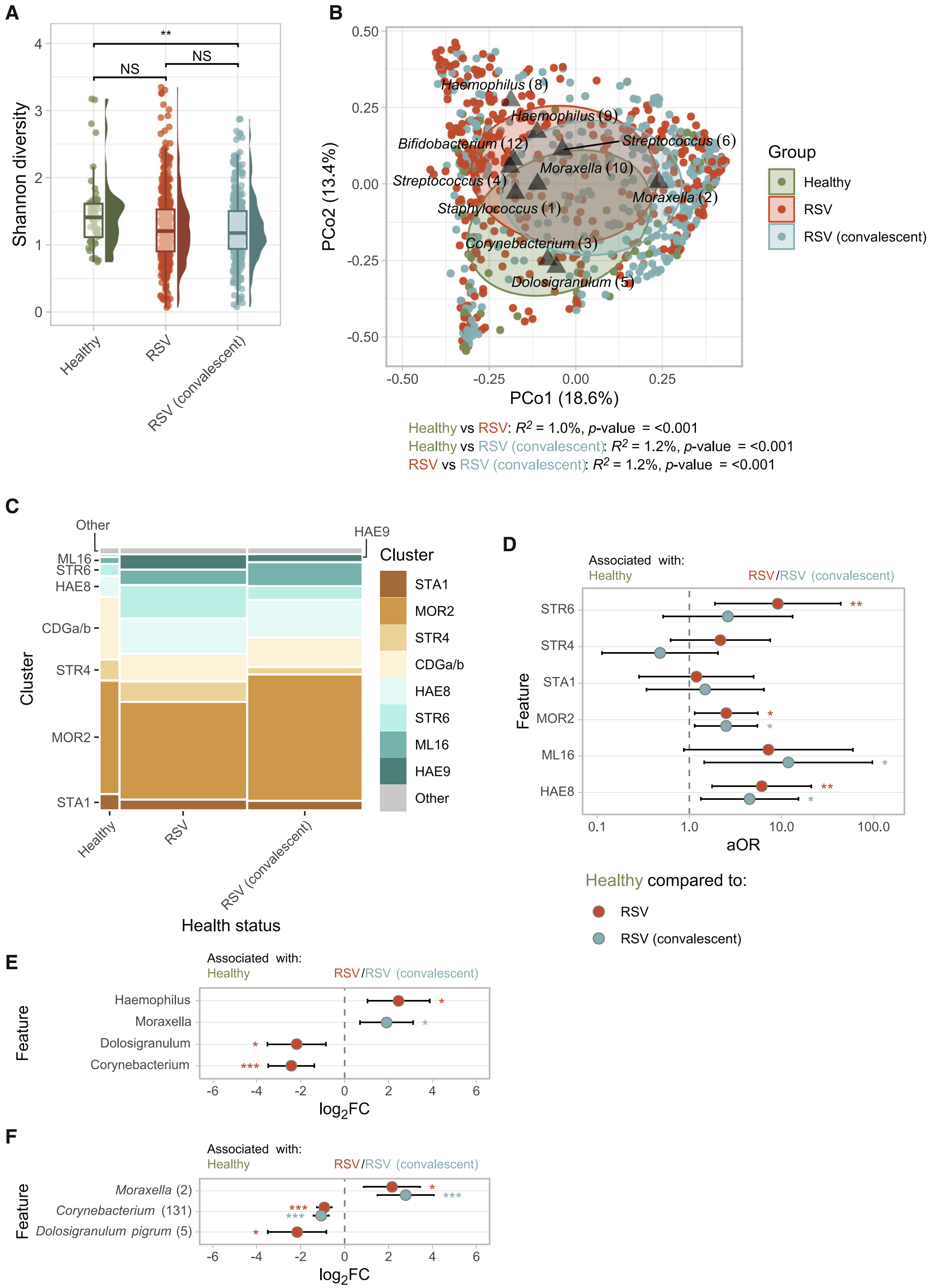

Figure 2. Microbiota diversity, cluster membership, and composition during RSV infection and at convalescence

(A) ASV-level Shannon diversity (non-rarefied) between study groups. Boxplots represent the 25th and 75th percentiles (lower and upper boundaries of boxes, respectively), the median (middle horizontal line), and measurements that fall within 1.5 times the interquartile range (IQR; distance between 25th and 75th

(legend continued on next page)

CellPress
OPEN ACCESS

Using logistic mixed-effects regression analysis (including cluster, age, and gender as fixed effects and study site as random effect), we found that the HAE8, STR6, and MOR2 clusters were associated with RSV infection (adjusted odds ratio [aOR] 6.1, 9.1, and 2.52, respectively, all $p < 0.02$) when compared to the CDGa/b cluster (Figure 2D). The smaller HAE9 cluster ($n = 28$, 3.7%) was even exclusively observed in cases. Similar, but less outspoken, cluster distributions were found at convalescence, although the association with *S. pneumoniae*/STR6 cluster disappeared, suggesting that this cluster is more strongly associated with the acute phase of infection.

Differentially abundant taxa in healthy controls, in infants during RSV infection, and during convalescence

Differential abundance analyses at genus level (MaAsLin2) largely confirmed our cluster-based analyses, with a higher relative abundance of *Haemophilus* spp. (adjusted for age, gender, and study site [random effect]; $\log_2$FC = 2.45, *q* value = 0.017) and lower abundance of *Corynebacterium* and *Dolosigranulum* spp. during RSV infection ($\log_2$FC = −2.43 and −2.18, respectively, both *q* value ≤ 0.021). Interestingly, *Moraxella* spp. were more abundant at RSV convalescence when compared to healthy controls (Figures 2E and 2F; $\log_2$FC = 1.91, *q* value 0.023). Since we adjusted for age in these models, this latter finding might indicate a long-lasting effect of viral infection, inflammation, and/or (antibiotic) treatment on the bacterial microbiota.

Bacterial microbiota in relation to the severity of RSV infection

Next, we stratified RSV infection according to severity e.g., mild (RESViNet score 0–7; $n = 218$), moderate (8–13; $n = 106$), and severe disease (14–20; $n = 47$).[22] Despite no differences in alpha-diversity or bacterial density between severity groups and healthy controls (Figures 3A and S2B, respectively), we found large differences in overall microbial community composition. PCoA visualization indicated that the deviation from health became larger with increasing severity (Figure 3B). Indeed, a stepwise increase in proportion of microbial community variance was explained by disease severity (PERMANOVA; R^2 = 1.3%, 3.0%, and 8.1% for mild, moderate, and severe RSV compared to healthy controls, respectively; *p* value ≤ 0.003). Additionally, the microbial "dissimilarity" within individuals, when comparing communities during RSV infection with those during convalescence, was significantly higher for cases with severe RSV when compared to mild disease (linear mixed-effects model corrected for age, gender, time between infection and convalescence, and study site; $\beta = 0.152$, *p* value = 0.003; Figure 3C).

Clustering and differential abundance between mild, moderate, and severe RSV infection

In line with previous analyses, cluster membership and differential abundance analyses indicated increasing deviations from health with increasing severity of RSV infections. Especially STR6 (likely *S. pneumoniae*), HAE8, and STR4 clusters were enriched in severe RSV cases (compared to CDGa/b; logistic mixed-effects model; aOR 45.1, 45.7, and 16.1, respectively, $p < 0.03$; Figure 3D). Differential abundance analyses confirmed that health-associated microbes including *Corynebacterium* and *Dolosigranulum* spp. became more severely depleted with increasing RSV disease severity (*Corynebacterium* spp. $\log_2$FC = −1.90, −2.71, and −3.70 for mild, moderate, and severe RSV, respectively, *q* value ≤ 0.005; Figures 3E and 3F). To rule out confounding by cohort, we repeated these tests for the case-control cohort only, which showed highly similar results (data not shown).

Microbiota deviations following RSV infection

Additionally, we wondered if we could detect "microbial scars" during RSV convalescence, speculating that residual microbial deviations would be more extreme following more severe disease. Using ASV-level differential abundance analyses (MaAsLin2), we found limited support for this hypothesis, with only several rare *Corynebacterium* spp. (18, 81, and 131) being associated with previous severe RSV infection (compared to previous mild disease; *q* value = 0.165; *p* value ≤ 0.006). After correction for use of antibiotics during infection, these differences were no longer significant. We also tested whether symptoms (blocked nose/cough/wheeze) during convalescence were related to remaining microbiota perturbations. We indeed found that persistence of respiratory symptoms were associated with a high relative abundance of *Haemophilus* and lack of *Dolosigranulum* (linear mixed-effects model including the covariates (remaining)

percentiles; whiskers). Statistical significance was assessed using linear mixed-effects models with Shannon diversity as outcome, age, gender, sequencing depth (scaled), and health status (healthy controls, RSV, or RSV convalescent) as fixed effects and study site as random effect. Time between RSV infection and convalescence (fixed effect) and subject ID were additionally included for comparisons between RSV and RSV convalescence.

(B) Principal coordinate analysis (PCoA) based on Bray-Curtis dissimilarities showing the nasopharyngeal microbiota composition in healthy controls ($n = 52$), acute RSV ($n = 374$), and (matched) samples collected at convalescence ($n = 338$). Percentages in brackets denote the total variance explained by the first two principal coordinates. Each data point (dot) indicates a nasopharyngeal sample colored by study group. Ellipses denote the standard deviation of data points for each group. The 10 highest ranking ASVs over the first days of life were simultaneously visualized (triangles). R^2 and statistical significance of the association between health status and the overall microbiota composition was assessed using PERMANOVA tests (1,000 permutations, adjusting for age, gender, and study site [restricted permutations]).

(C) Mosaic plot showing cluster membership in healthy controls, acute RSV, and convalescent samples.

(D) Adjusted odds ratios (aORs) for cluster membership during RSV infection or convalescent phase (categorical variable; predictor), adjusted for age, gender (fixed effects), and study site (random effect), with health status as an outcome variable. Two models were simultaneously visualized: model (1) RSV infection vs. health and model (2) RSV convalescence vs. health. HAE9 cluster was not visualized, as it was highly prevalent during both RSV infection and convalescence, but absent in healthy controls. Whiskers denote 95% confidence intervals (CIs; Wald method). Asterisks denote statistical significance (NS, not significant [$p > 0.05$]; *, $p \leq 0.05$; **, $p \leq 0.01$; ***, $p \leq 0.001$).

(E and F) Log_2 fold change (FC) of features (genera [E]/ASVs [F]) based on MaAsLin2 (linear mixed-effects model) with health status (healthy controls, RSV infection, and convalescence) as variable of interest, adjusted for age, gender (fixed effects), and study site (random effect) and $\log_2$-transformed relative abundance as outcome. Only features present in ≥5% of samples at >0.1% relative abundance were tested. ASVs with a $q \leq 0.05$ are depicted. Whiskers denote 95% confidence intervals (CIs; Wald method). Asterisks denote statistical significance (*, $q \leq 0.05$; **, $q \leq 0.01$; ***, $q \leq 0.001$).

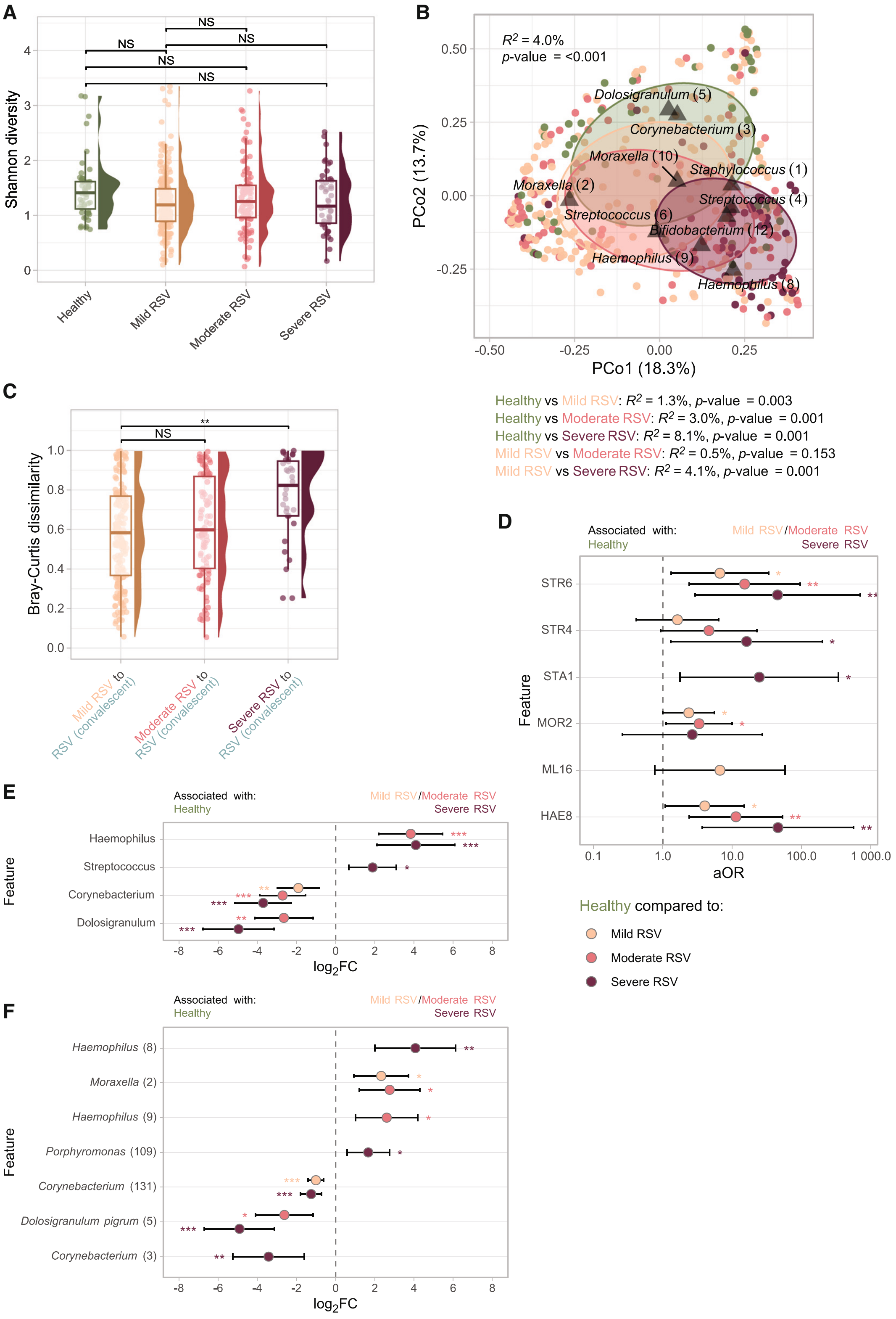

(legend on next page)

respiratory symptoms, age at recovery, time since RSV infection, and gender [fixed effects] and study site [random effect]; $\log_2$FC = 1.70, q value = 0.029 and $\log_2$FC = −1.47, q value = 0.056, respectively; Figure 4).

Early-life microbiota diversity and composition

Last, we decided to assess whether, already in early life, microbial community development was related with consecutive RSV susceptibility and severity. We first characterized the microbiota of birth cohort participants during the first 11 days of life. When assessing alpha-diversity on ASV level, we found a negative log-linear relationship between age of sampling and microbial diversity (Figure 5A; linear mixed-effects model; adjusted for age, gender, siblings, sequencing depth, and study site; $\beta = -0.217$, p value = 0.011), indicating alpha-diversity decreased with age over the first 12 days of life. Inversely, $\log_{10}$-transformed bacterial density showed a positive log-linear relationship with age (Figure S2C; $\beta = 0.595$, p value = 2.7×10^{-8}).

Next, we assessed the overall microbiota community composition, showing again that age category had a significant impact on microbiota composition (n = 772; PERMANOVA; permutations restricted within study site; R^2 = 5.0% p value < 0.001; Figure 5B). Excluding samples collected from Spanish infants, who were generally younger (and therefore prevented proper correction for day of sampling alongside study site in our models), we could still detect a significant age effect (n = 662; PERMANOVA; R^2 = 1.1%, p value < 0.001), confirming that this effect was not caused by differences between study sites. Apart from age, other clinical factors were also associated with differences in the overall microbial community composition. Using bivariable PERMANOVA tests (adjusting for age category and restricting permutations within study site where appropriate), we found large effects of study site (R^2 = 2.2%, p value < 0.001) and modest but significant effects of mode of delivery (R^2 = 0.9%, p value = 0.002) and having siblings (R^2 = 0.8%, p value < 0.001) on the overall microbial community composition. There was no significant effect of season of birth, gender, or feeding type.

Using PCoA, we found an especially clear separation between samples from infants at the age of <5 days compared to infants aged ≥5 days (Figure 5B). Overall, at baseline, we mainly observed clusters characterized by *Staphylococcus* (1) (STA1; n = 440, 57%), STR4 (n = 194, 25%), CDGa/b (n = 90, 12%), and MOR2 (n = 30, 3.9%). STR4-cluster membership was more prevalent in infants <5 days when compared to older infants (53% vs. 15%, two-sided Fisher's exact test, p value < 0.001), whereas STA1 and CDG clusters were enriched in older children (39% vs. 64% and 3.8 vs. 15%, in <5 days and >5 days, respectively; p value < 0.001; Figure 5C). Although generally less prevalent, MOR2-cluster membership was more frequent in children >5 days of life (4.6% vs. 1.9% in <5 days, p value = 0.082).

We investigated whether these early-life microbiota profiles were associated with the chance of consecutive RSV infection (independent of severity) during the first year of life. We found no significant association between the overall microbial community composition at baseline and the subsequent occurrence of RSV (PERMANOVA; adjusted age of sampling, gender, and season of birth; permutations restricted within study site; R^2 = 0.1%, p value = 0.278). Additionally, we found that none of the microbial community profiles were associated with the likelihood of an RSV infection (logistic mixed-effects regression accounting for age, gender, siblings, season, and study site; p value > 0.05).

Early-life microbiota and severity of RSV later in life

We hypothesized that early-life microbiota profiles could predict the severity of consecutive RSV infections in the first year of life. For this analysis, we included only baseline microbiota profiles of infants who had a proven RSV infection in the first year of life (N = 197). Since these RSV infections were generally mild (RESViNet score 0–7; N = 173, 89%), we considered seeking medical attendance as a measure to discriminate between more and less severe disease (N = 85 and N = 100 for medically attended and not medically attended RSV infections, respectively). Infants suffering from a medically attended RSV infection were younger when compared to infants without medically attended RSV infection (two-sided Student's t test; $\beta = -32.8$, p value = 0.022). We therefore corrected for age at RSV infection in all downstream analyses.

The overall baseline microbial community composition, adjusted for age of sampling, age at RSV infection, gender, season of birth, and study site (permutation block), showed a trend toward a significant correlation with severity of disease (medically

Figure 3. Associations between microbiota diversity, stability and composition, and RSV infection severity

(A) ASV-level Shannon diversity (non-rarefied) in healthy controls (n = 52) compared to mild (RESViNet score 0–7; n = 218), moderate (8–13; n = 106), and severe RSV (14–20; n = 47). Statistical significance was assessed using linear mixed-effects models with Shannon diversity as outcome, age, gender, sequencing depth (scaled), and RSV severity (healthy, mild, moderate, and severe RSV) as fixed effects and study site as random effect.

(B) Principal coordinate analysis (PCoA) depicting the overall nasopharyngeal microbiota composition in healthy controls, mild, moderate, and severe RSV. The R^2 and statistical significance across all severity groups was estimated and depicted in the upper left corner. Pairwise differences between groups were additionally modeled, adjusting for age, gender, and study site (restricted permutations).

See legend Figure 2B.

(C) Bray-Curtis dissimilarity between paired RSV infection and convalescent samples, stratified by mild, moderate, and severe disease (n = 179, n = 89, and n = 32, respectively). Statistical significance assessed using a linear mixed-effects model with severity as outcome of interest, adjusted for age at RSV infection, gender, and time between RSV infection (fixed effects) and study site (random effect), and Bray-Curtis dissimilarity as outcome.

(D) Adjusted odds ratios (aORs) for cluster membership during mild, moderate, or severe RSV infection (categorical variable; predictor), adjusted for age, gender (fixed effects), and study site (random effect), with severity as an outcome variable. Three logistic mixed-effects regression models were simultaneously visualized, comparing cluster membership in (1) mild RSV vs. health, (2) moderate RSV vs. health, and (3) severe RSV vs. health. Although the HAE9 cluster was not visualized, it was highly associated with RSV infection and convalescence, as it was absent in healthy controls. Whiskers denote 95% confidence intervals (CIs; Wald method). Asterisks denote statistical significance (NS, not significant [$p > 0.05$]; *, $p \leq 0.05$; **, $p \leq 0.01$; ***, $p \leq 0.001$).

(E and F) Log_2 fold change (FC) of features (genera [E]/ASVs [F]) based on MaAsLin2 (linear mixed-effects model) with RSV severity (compared to healthy controls) as variable of interest, adjusted for age, gender (fixed effects), and study site (random effect) and $\log_2$-transformed relative abundance as outcome. Only features present in ≥5% of samples at >0.1% relative abundance were tested. Asterisks denote statistical significance (*, $q \leq 0.05$; **, $q \leq 0.01$; ***, $q \leq 0.001$).

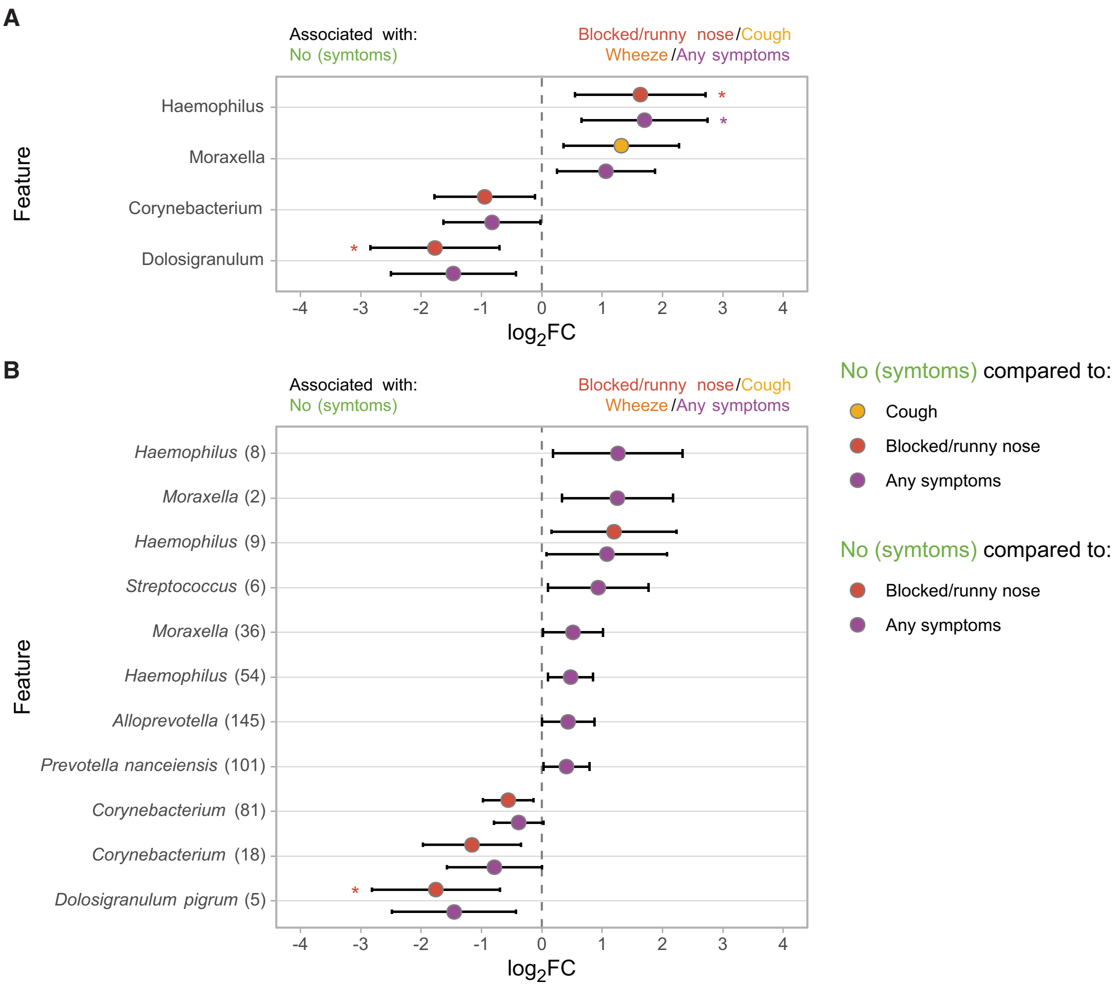

Figure 4. Associations between microbiota profiles at RSV convalescence and remaining symptoms

(A and B) Log_2 fold change (FC) of features (genera [A]/ASVs [B]) based on MaAsLin2 (linear mixed-effects model) with symptoms (yes/no cough, wheeze, blocked/runny nose, and any symptoms) as variable of interest, adjusted for age, gender, and time between RSV infection and convalescence (fixed effects) and study site (random effect) and log_2-transformed relative abundance as outcome. Only features present in $\geq$5% of samples at >0.1% relative abundance across RSV and RSV convalescence samples were tested. Whiskers denote 95% confidence intervals (CIs; Wald method). Asterisks denote statistical significance (*, $q \leq 0.05$; **, $q \leq 0.01$; ***, $q \leq 0.001$).

attended RSV infection, yes/no; PERMANOVA; R^2 = 0.8%, *p* value = 0.107). Baseline microbiota clusters were more clearly associated with consecutive RSV severity, with a lower rate of MOR2 cluster (vs. CDG) in those infants who developed a medically attended RSV infection (logistic mixed-effects regression model with age at sampling, age at infection, study site, and birth season as covariates; aOR [95% confidence interval (CI)] = 0.144 [0.022–0.938], *p* value 0.022; Figure 5D).

To further quantify the association between baseline microbiota features and severity of (mostly mild) RSV infection, we fitted a random forest classification model with 39 microbial features (selected because they were present in >5% of samples at $\geq$0.1% relative abundance) as "predictors," including age at the moment of RSV infection as a covariate and considering medical attendance yes/no as the outcome (*N* = 137 infants; only including samples collected $\geq$5 days of age). After model tuning, we found only a classification power with an area under the curve (AUC) of 0.59 (95% CI 0.49–0.69) based on out-of-bag predictions (permutation test *p* value = 0.087), with similar results based on 5-fold cross-validated predictions (Figure 5E). Shapley additive explanations (SHAP) values, representing the impact on the model output, were calculated to assess variable importance. Mean absolute SHAP values indicated, in line with previous findings, that *Dolosigranulum pigrum* (5) is most important in discerning medically versus not medically attended RSV, followed by *Staphylococcus* (1). Age at first RSV infection, although associated with medical attendance, only showed mediocre importance (ranking 4th; Figures 5F and 5G). Overall, this classification model showed a preliminary and modest distinction between medical attendance and non-medical attendance during RSV based on the baseline microbiome, though it should be noted that severe cases were mostly lacking in this cohort.

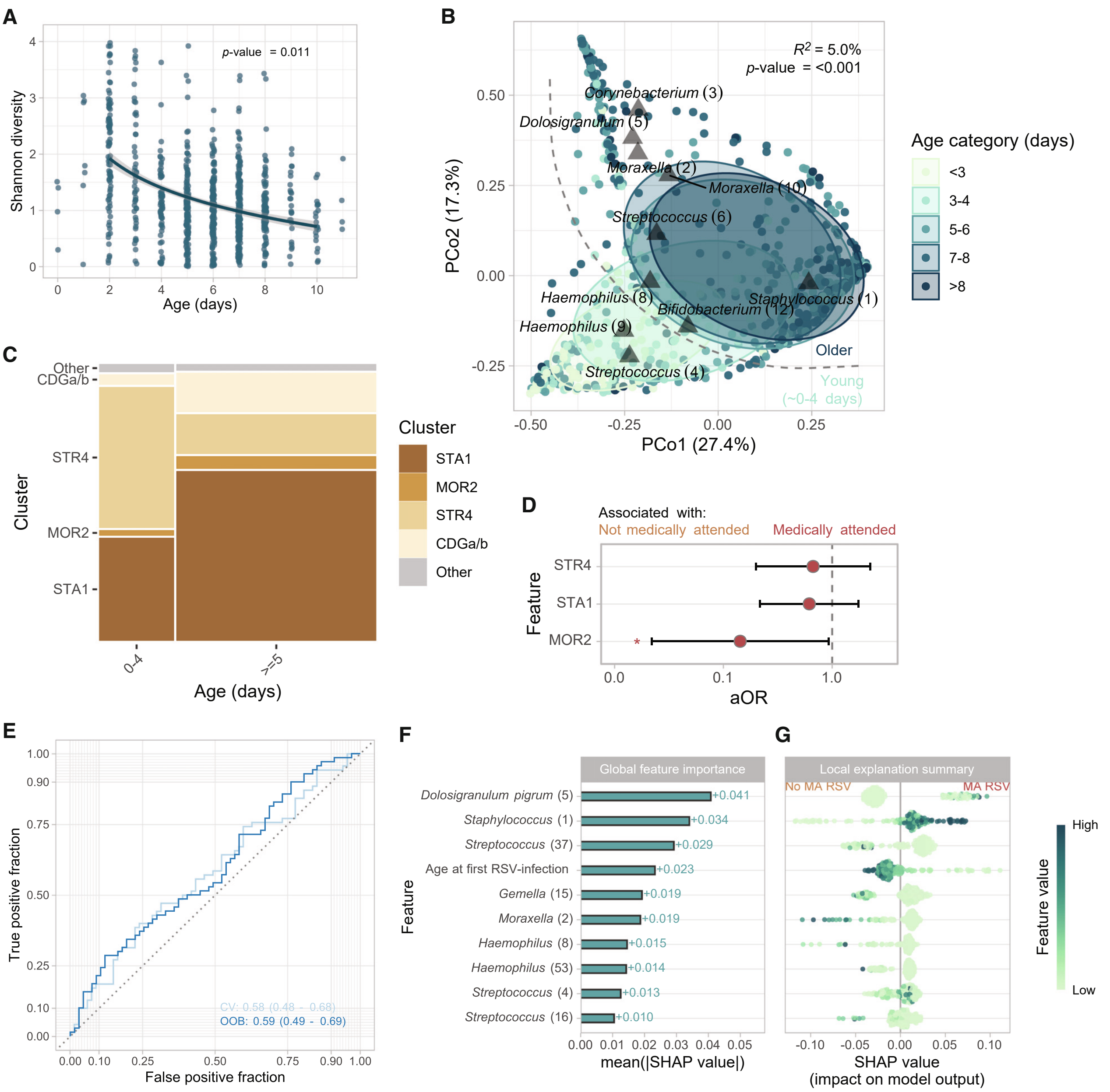

Figure 5. Early-life microbiota development and cluster membership

(A) ASV-level Shannon diversity (non-rarefied) for baseline samples over age. Statistical significance was assessed using a linear mixed-effects model, including Shannon diversity as outcome, the natural log of age, sequencing depth, season of birth, gender, and presence of siblings as fixed effects and study site as random effect.

(B) Principal coordinate analysis based on Bray-Curtis dissimilarities showing the nasopharyngeal microbiota composition over the first days of life (<3 days, n = 97; 3–4 days, n = 113; 5–6 days, n = 256; 7–8 days, n = 255; >8 days, n = 51). The R^2 and statistical significance of age categories was estimated (PERMANOVA; restricted permutations within study site) and depicted in the upper right corner.

See legend Figure 2B.

(C) Mosaic plot showing cluster membership over the first days of life, stratified by age (<5 days vs. ≥5 days). Only clusters including at least 2% of samples are shown (CDGa/b, STR4, STA1, and MOR2 clusters).

(D) Adjusted odds ratios (aORs) for cluster membership at baseline (categorical variable; predictor), adjusted for age at sampling, adjusted for age at first RSV infection, gender, season of birth, presence of siblings (fixed effects), and study site (random effect), with medically attended RSV infection yes/no as an outcome variable. Whiskers denote 95% confidence intervals (CIs; Wald method). Asterisks denote statistical significance (NS, not significant [$p > 0.05$]; *, $p \leq 0.05$; **, $p \leq 0.01$; ***, $p \leq 0.001$).

(E) Area-under-the-curve (AUC) receiver operating curves (ROCs) to evaluate the random forest classifier to discriminate between medically attended ($N = 85$) and not medically attended RSV ($N = 100$). The model includes 39 ASVs and age (in days) at first RSV infection as predictors. Curves were calculated for out-of-bag

(legend continued on next page)

A similar model (without age at first RSV infection), using a much larger sample size (n = 562) to predict RSV occurrence within the first year of life, performed worse (AUC [95% CI] = 0.54 [0.48–0.59]; permutation test p value = 0.235). Together, these findings indicate that getting an RSV infection in the first year of life is stochastic, though severity may be modestly related to early-life respiratory microbiota.

DISCUSSION

Using two large RSV infant cohorts from within the RESCEU consortium, we were able to assess the relationship between the respiratory microbiome and disease phenotype across the full scale of RSV severity. In addition, we studied the respiratory microbiome in the first 11 days of life and assessed their association with RSV susceptibility and severity over the first year of life. We found that stronger microbial deviations from a healthy microbiota composition co-occurred with increasing severity of disease. Although no association between the respiratory microbiome and the risk of any type of symptomatic RSV infection was found, we found a weak association between the respiratory microbiome in early life and the severity of consecutive RSV infection.

In this study, we found that stronger microbial deviations from health co-occurred with increasing severity of RSV infection. Lower abundance of *Dolosigranulum* and *Corynebacterium* and higher abundance of *Streptococcus*, *Haemophilus*, and *Moraxella* were associated with more severe infection, whereas higher abundance of *Dolosigranulum* and *Corynebacterium* were associated with health. These findings are in line with previous studies, describing that *S. pneumoniae*, *Moraxella*, and *Haemophilus* were associated with more severe disease.[12,13,16] However, these studies were mostly conducted in patients with medically attended RSV infections, thus excluding the very mild cases. In this study, we found that even in very mild RSV infections, the bacterial upper respiratory tract microbiome differs from that of healthy individuals.

The associations between the microbial community composition and disease severity may represent either bacterial co-stimulation of inflammation or the direct interaction between respiratory bacteria and RSV. A genome-wide association study performed on case-control study samples used included in this study found that specifically genes relevant to neutrophil trafficking and cytoskeletal functions were associated with more severe RSV infections.[19] Previous studies have described interactions between the respiratory microbiome, immune responses, and RSV severity. Among others, it has been shown that *H. influenzae* and *Streptococcus*-dominated profiles were related to more severe infections and that they were also related to heightened expression of genes related to Toll-like receptors and neutrophil and macrophage activity.[11] In line, it has been shown that *Haemophilus* has been associated with higher levels of CXCL8, a chemokine indicative of disease severity.[15,23] Others have shown that bacterial small RNAs from bacteria associated with bronchiolitis were more abundantly present in RSV bronchiolitis compared to rhinovirus bronchiolitis and were associated with a relative upregulation of IL-6 and IL-8 pathways and downregulation of IL-17A pathways, thus promoting proinflammatory responses.[24]

A targeted meta-genomics study on the RSV samples included in this study found that viral co-detection was observed in 26% of the children. Interestingly, this was not associated with clinical outcomes.[25] Although viral co-detection does not seem to have effects on the severity of RSV infection, several studies have shown that bacterial pulmonary co-infection is not uncommon in children with a severe RSV infection.[26,27] Together with a potential mediating role of the bacterial microbiota in inflammation, and thereby the severity of disease, this would argue for careful decision-making regarding RSV disease that is generally considered of pure viral etiology. There is some evidence that, in children requiring mechanical ventilation, clinical outcomes are improved by early antibiotic treatment.[28] However, in line with the microbiological findings of our study, there is no compelling evidence that antibiotic treatment may improve clinical outcomes in mild or moderately non-critically ill children.

Additionally, we found that, although the microbial community composition at 6–8 weeks after infection had shifted partially toward that of a “healthy” profile, there was still an association with residual symptoms. The microbial signature related to remaining symptoms is reminiscent of the signal associated with more severe RSV (yet not directly related to RSV severity), possibly hinting toward a broad modulating impact of the bacterial respiratory microbiota on respiratory disease symptoms.

Again, higher abundance of *Haemophilus* at convalescence was associated with residual respiratory symptoms, after the initial infection was cleared. Though we were unable to find other studies looking at the association between the convalescent microbiome and residual complaints, the partial recovery of the microbiome to a “normal” composition has been previously observed in young children recovering from severe respiratory infections.[29] In this study, the residual differences with the control group were mostly found in a higher abundance of *Streptococcus anginosus* and gram-negative bacteria like *Porphyromonas* spp. and *Neisseria* spp, whereas we found more children with a *Haemophilus* and *Moraxella*-dominated profile (14). This difference might well be explained by the fact that we included only children with RSV infection, instead of a mix of respiratory infections, and we looked at a broad range of severity, instead of only severely ill children. Several studies have associated a high abundance of *Haemophilus* with asthma

(OOB) and 5-fold cross-validated (CV) predictions, giving similar results, validating the use of OOB estimates for subsequent analyses. 95% Confidence intervals were calculated using the DeLong method as implemented in the *pROC* package.

(F) Mean absolute permutation-based Shapley additive explanations (SHAP) values of the 10 most important features of the model described in (E).

(G) SHAP local explanation summary plot with individual SHAP values for each subject (N = 137 per feature). Each dot has three characteristics: (1) vertical location indicates the feature it is depicting, (2) the color shows whether that feature was high or low for a given subject (scaled relative abundance or age), and (3) horizontal location shows whether that value caused a higher or lower prediction. Higher SHAP values indicate a positive contribution to the likelihood of developing a medically attended RSV infection.

CellPress
OPEN ACCESS

and wheezing.[30–32] Notably, one study noted that this association could only be found in children who had been allergically sensitized at an early age, again indicating unknown interactions between the microbiome and the host immune system.[33] Furthermore, a high abundance of *Moraxella* and *Klebsiella* during RSV infection was associated with recurrent wheezing, indicating that these differences in microbial community composition and immune response may be associated with long-lasting effects.[15] After further follow-up of this cohort, it will be interesting to investigate whether the recovery of the microbiome after infection, and potentially the (persistent) abundance of *Haemophilus*, could be a biomarker for later development of wheeze and asthma.

Lastly, the combination of early-life sampling and longitudinal detailed surveillance of RSV in the birth cohort study offered the unique opportunity to study the association between the early-life microbiome occurrence and severity of RSV infection during the first year of life. From the 773 infants who were sampled shortly after birth, nearly 200 children experienced an RSV infection during the first year of life. We only sampled infants during respiratory symptoms, meaning that asymptomatic infections may have been missed in this study. We found no association between the baseline respiratory microbial community composition and the occurrence of RSV during the first year of life. A recent seroconversion study from the Netherlands showed that the majority of children have experienced an RSV infection before the age of 2 years, and that by 5 years of age, nearly all children have contracted RSV at least once.[34] Based on these findings, we hypothesize that the timing of first RSV infection is mainly driven by viral exposure and decline of maternal antibodies, and not by (very) early-life microbial community composition.

We found no association between the early-life microbial community composition and risk of RSV infection, and only a modest association with the severity of RSV disease. This is contradictory to previous studies showing an association between the respiratory microbial community composition and timing of first infection or frequency of respiratory infection.[6–9,35] These studies, however, did not look at RSV infections specifically, but at respiratory infections in general. Additionally, a study by Grier et al., which did focus on RSV infections, found in a longitudinal case-control study different abundances of keystone species prior to infection in children with and without infection.[10] Baseline samples were collected at the age of 1 month, which is later than in our study. However, these results were based on only 12 children with RSV. Reasons why this association was less clear in our cohort are as follows: (1) the group of children followed from birth developed mostly mild, and occasionally moderately severe, RSV infections, which limits the power to detect differences across the severity spectrum; and (2) the neonatal samples were collected between the first day of life and day 11 after birth, which may be too early to detect more persistent colonization patterns that could affect RSV susceptibility. This is in line with previous results from one of our cohorts finding low-density, very transient signals that may not reflect actual colonization in the first week of life.[7] A study by Vissing et al., for example, found an association with the carriage of respiratory pathobionts at the age of 1 month, and subsequent development of pneumonia or bronchiolitis.[6] We therefore recommend more frequent longitudinal sampling in future studies in order to provide more in-depth information regarding the microbial community composition preceding RSV infection, in relation to susceptibility and severity of disease.

Limitations of the study

Limitations of this study include the use of 16S-rRNA-based sequencing methods, which are often insufficient to identify bacteria up to species level. Though technically a necessity due to the low biomass of nasopharyngeal samples, this lack of species-level information is unfortunate, as it is well known that different species from the same genus can have vastly different effects in the respiratory tract, making it difficult to hypothesize about the biological mechanisms underlying these findings. Additionally, we have no serological data to detect any asymptomatic infections in our cohort during the first year of life. Studies have shown that asymptomatic infections do occur more frequently than previously thought.[36] This may especially have affected the resolution of the analyses on the association between the early-life microbiome and subsequent RSV severity. In our analysis of microbiota differences between infection and convalescence, we adjusted for age in the models. However, due to the longitudinal study setup, all convalescent samples were collected at a later age compared to the matching infection sample from the same child, complicating age correction. Last, due to a limited sample size, we could only internally validate our classification model; preferably, this would have been done using a separate validation cohort.

Conclusion

During RSV infection, we observed that RSV disease severity was associated with greater deviation in microbial community profiles when compared to healthy children. Importantly, also during the recovery phase, the respiratory microbiome was linked with the presence of residual respiratory symptoms. Further follow-up can shed light on the relation between these microbial changes during and after RSV infection and the later development of wheeze or asthma. The early-life respiratory microbial community composition seems unrelated to the risk of RSV infection, which may be more driven by risk factors like age at RSV, season, and crowding. Importantly, we did detect a modest association between the early-life microbiome and the severity of consecutive RSV infection, suggesting that the early-life microbial community may in part modulate viral infection severity.

RESOURCE AVAILABILITY

Lead contact

Further information and requests for resources and reagents should be directed to and will be fulfilled by the lead contact, Debby Bogaert (d.bogaert@ed.ac.uk).

Material availability

This study did not generate new unique reagents.

Data and code availability

- 16S-rRNA sequencing data (paired FASTQ files) have been deposited at the NCBI Sequence Read Archive database publicly available as of the date of publication. Bioproject: PRJNA914884.

- This paper does not report original code. A release version of the code to process and analyze the data has been archived in a Zenodo repository and is publicly available as of the date of publication (https://doi.org/10.5281/zenodo.13941981). DOIs are listed in the key resources table.
- Any additional information required to reanalyze the data reported in this paper is available from the lead contact upon request.

CONSORTIA

The members of the RESCEU Investigators are Philippe Beutels, Jeroen Aerssens, Bishoy Rizkalla, Thea Kølsen Fischer, Terho Heikkinen, Charlotte Vernhes, Scott Gallichan, Carlo Giaquinto, Joanne Wildenbeest, Marie-Noelle Billard, Roy Zuurbier, Koos Korsten, Marlies van Houten, Annefleur Langedijk, Peter van de Ven, Louis Bont, Maarten van den Berge, Adam Meijer, Ana Dacosta-Urbieta, Irene Rivero-Calle, Alberto Gómez-Carballa, Sara Pischedda, Carmen Rodriguez-Tenreiro, Federico Martinón-Torres, Eva Molero, Simon Drysdale, Joseph McGinley, Gu-Lung Lin, Matthew Snape, Andrew Pollard, Andrew Ives, Helen Wolfenden, Sanjay Salgia, Rohoth Shetty, Steve Cunningham, Harish Nair, Harry Campbell, Thom O'Neill, Margaret Miller, Julie Baggott, Catherine Beveridge, Rachael McKernan, Peter Openshaw, Michael Abram, Kena Swanson, and Veena Kumar.

ACKNOWLEDGMENTS

We thank all children and parents who participated in this study. This work was supported in part by the Innovative Medicines Initiative 2 Joint Undertaking (grant 116019), the Netherlands Organisation for Scientific Research (NWO-VIDI; grant 91715359), and NHS Research Scotland/the Chief Scientist Office CSO/NRS (SCAF/16/03). The providers of funding of these studies had no involvement in the study design, study execution, data analysis, or reporting of the data.

AUTHOR CONTRIBUTIONS

D.B. and L.B. designed the experiments. J.W., R.P.Z., M.A.v.H., S.C., M.S., S.B.D., R.S.T., F.M.-T., P.J.M.O., J.A., L.B., and A.J.P. were responsible for (supervision of) participant enrollment and sample and data collection. R.H., K.A., and M.L.J.N.C. were responsible for laboratory processing of samples. W.A.A.d.S.P. performed bioinformatic processing and M.K. and W.A.A.d.S.P. performed statistical analyses. W.A.A.d.S.P., M.K., and D.B. wrote the paper. All authors significantly contributed to interpretation of the results, critically revised the manuscript for important intellectual content, and approved the final manuscript.

DECLARATION OF INTERESTS

D.B. received funding from OM Pharma and GlaxoSmithKline. F.M.-T. declares that his institution received payment from GSK, Ablynx, Abbot, Seqirus, Sanofi, MSD, Merck, Pfizer, Roche, Regeneron, Janssen, MedImmune, Novavax, Novartis, and GSK for vaccine trials; F.M.-T. also reports receiving honoraria for lectures from Sanofi, MSD, Moderna, GSK, Biofabri, AstraZeneca, Novavax, Janssen, and Pfizer; payment of travel expenses and meeting fees from Pfizer, MSD, GSK, and Sanofi; and participation on data safety monitoring boards or advisory boards for Pfizer, GSK, Moderna, Sanofi, AstraZeneca, and Biofabri. J.W. has been an investigator for clinical trials sponsored by pharmaceutical companies including AstraZeneca, Merck, Pfizer, Sanofi, and Janssen with all funds paid to University Medical Center Utrecht (UMCU) and has participated in the advisory boards of Janssen and Sanofi with fees paid to UMCU.

STAR★METHODS

Detailed methods are provided in the online version of this paper and include the following:

- KEY RESOURCES TABLE
- EXPERIMENTAL MODEL AND SUBJECT DETAILS
  - Study population and sample collection
  - Demographic and clinical data
- METHOD DETAILS
  - Bacterial DNA isolation
  - Bacterial DNA quantification
  - 16S-rRNA-sequencing
  - Bioinformatic processing 16S-rRNA sequences
  - Contamination detection and removal
- QUANTIFICATION AND STATISTICAL ANALYSES
  - Comparisons
  - Modeling approach
  - Alpha-diversity analysis
  - Beta-diversity analysis
  - Clustering
  - Differential abundance analysis
  - Random forest modeling

SUPPLEMENTAL INFORMATION

Supplemental information can be found online at https://doi.org/10.1016/j.xcrm.2024.101836.

Received: July 2, 2024
Revised: August 26, 2024
Accepted: November 4, 2024
Published: December 5, 2024

REFERENCES

1. Shi, T., McAllister, D.A., O'Brien, K.L., Simoes, E.A.F., Madhi, S.A., Gessner, B.D., Polack, F.P., Balsells, E., Acacio, S., Aguayo, C., et al. (2017). Global, regional, and national disease burden estimates of acute lower respiratory infections due to respiratory syncytial virus in young children in 2015: a systematic review and modelling study. Lancet *390*, 946–958. https://doi.org/10.1016/S0140-6736(17)30938-8.
2. Figueras-Aloy, J., Manzoni, P., Paes, B., Simões, E.A.F., Bont, L., Checchia, P.A., Fauroux, B., and Carbonell-Estrany, X. (2016). Defining the Risk and Associated Morbidity and Mortality of Severe Respiratory Syncytial Virus Infection Among Preterm Infants Without Chronic Lung Disease or Congenital Heart Disease. Infect. Dis. Ther. *5*, 417–452. https://doi.org/10.1007/s40121-016-0130-1.
3. Checchia, P.A., Paes, B., Bont, L., Manzoni, P., Simões, E.A.F., Fauroux, B., Figueras-Aloy, J., and Carbonell-Estrany, X. (2017). Defining the Risk and Associated Morbidity and Mortality of Severe Respiratory Syncytial Virus Infection Among Infants with Congenital Heart Disease. Infect. Dis. Ther. *6*, 37–56. https://doi.org/10.1007/s40121-016-0142-x.
4. Chaw, P.S., Hua, L., Cunningham, S., Campbell, H., Mikolajczyk, R., and Nair, H.; RESCEU Investigators (2020). Respiratory Syncytial Virus-Associated Acute Lower Respiratory Infections in Children With Bronchopulmonary Dysplasia: Systematic Review and Meta-Analysis. J. Infect. Dis. *222*, S620–S627. https://doi.org/10.1093/infdis/jiz492.
5. Verwey, C., Ramocha, L., Laubscher, M., Baillie, V., Nunes, M., Gray, D., Hantos, Z., Dangor, Z., and Madhi, S. (2023). Pulmonary sequelae in 2-year-old children after hospitalisation for respiratory syncytial virus lower respiratory tract infection during infancy: an observational study. BMJ Open Respir. Res. *10*, e001618. https://doi.org/10.1136/bmjresp-2023-001618.
6. Vissing, N.H., Chawes, B.L.K., and Bisgaard, H. (2013). Increased Risk of Pneumonia and Bronchiolitis after Bacterial Colonization of the Airways as Neonates. Am. J. Respir. Crit. Care Med. *188*, 1246–1252. https://doi.org/10.1164/rccm.201302-0215OC.
7. Bosch, A.A.T.M., de Steenhuijsen Piters, W.A.A., van Houten, M.A., Chu, M.L.J.N., Biesbroek, G., Kool, J., Pernet, P., de Groot, P.K.C.M., Eijkemans, M.J.C., Keijser, B.J.F., et al. (2017). Maturation of the Infant Respiratory Microbiota, Environmental Drivers, and Health Consequences. A

Prospective Cohort Study. Am. J. Respir. Crit. Care Med. *196*, 1582–1590. https://doi.org/10.1164/rccm.201703-0554OC.

8. Toivonen, L., Hasegawa, K., Waris, M., Ajami, N.J., Petrosino, J.F., Camargo, C.A., Jr., and Peltola, V. (2019). Early nasal microbiota and acute respiratory infections during the first years of life. Thorax *74*, 592–599. https://doi.org/10.1136/thoraxjnl-2018-212629.
9. Teo, S.M., Mok, D., Pham, K., Kusel, M., Serralha, M., Troy, N., Holt, B.J., Hales, B.J., Walker, M.L., Hollams, E., et al. (2015). The Infant Nasopharyngeal Microbiome Impacts Severity of Lower Respiratory Infection and Risk of Asthma Development. Cell Host Microbe *17*, 704–715. https://doi.org/10.1016/j.chom.2015.03.008.
10. Grier, A., Gill, A.L., Kessler, H.A., Corbett, A., Bandyopadhyay, S., Java, J., Holden-Wiltse, J., Falsey, A.R., Topham, D.J., Mariani, T.J., et al. (2021). Temporal Dysbiosis of Infant Nasal Microbiota Relative to Respiratory Syncytial Virus Infection. J. Infect. Dis. *223*, 1650–1658. https://doi.org/10.1093/infdis/jiaa577.
11. de Steenhuijsen Piters, W.A.A., Heinonen, S., Hasrat, R., Bunsow, E., Smith, B., Suarez-Arrabal, M.C., Chaussabel, D., Cohen, D.M., Sanders, E.A.M., Ramilo, O., et al. (2016). Nasopharyngeal Microbiota, Host Transcriptome, and Disease Severity in Children with Respiratory Syncytial Virus Infection. Am. J. Respir. Crit. Care Med. *194*, 1104–1115. https://doi.org/10.1164/rccm.201602-0220OC.
12. Rosas-Salazar, C., Tang, Z.Z., Shilts, M.H., Turi, K.N., Hong, Q., Wiggins, D.A., Lynch, C.E., Gebretsadik, T., Chappell, J.D., Peebles, R.S., Jr., et al. (2022). Upper respiratory tract bacterial-immune interactions during respiratory syncytial virus infection in infancy. J. Allergy Clin. Immunol. *149*, 966–976. https://doi.org/10.1016/j.jaci.2021.08.022.
13. Diaz-Diaz, A., Bunsow, E., Garcia-Maurino, C., Moore-Clingenpeel, M., Naples, J., Juergensen, A., Mertz, S., Wang, H., Leber, A.L., Gern, J., et al. (2022). Nasopharyngeal Codetection of Haemophilus influenzae and Streptococcus pneumoniae Shapes Respiratory Syncytial Virus Disease Outcomes in Children. J. Infect. Dis. *225*, 912–923. https://doi.org/10.1093/infdis/jiab481.
14. Raita, Y., Pérez-Losada, M., Freishtat, R.J., Hahn, A., Castro-Nallar, E., Ramos-Tapia, I., Stearrett, N., Bochkov, Y.A., Gern, J.E., Mansbach, J.M., et al. (2022). Nasopharyngeal metatranscriptome profiles of infants with bronchiolitis and risk of childhood asthma: a multicentre prospective study. Eur. Respir. J. *60*, 2102293. https://doi.org/10.1183/13993003.02293-2021.
15. Zhang, X., Zhang, X., Zhang, N., Wang, X., Sun, L., Chen, N., Zhao, S., and He, Q. (2020). Airway microbiome, host immune response and recurrent wheezing in infants with severe respiratory syncytial virus bronchiolitis. Pediatr. Allergy Immunol. *31*, 281–289. https://doi.org/10.1111/pai.13183.
16. Brealey, J.C., Chappell, K.J., Galbraith, S., Fantino, E., Gaydon, J., Tozer, S., Young, P.R., Holt, P.G., and Sly, P.D. (2018). *Streptococcus pneumoniae* colonization of the nasopharynx is associated with increased severity during respiratory syncytial virus infection in young children: S. pneumoniae detection in RSV infection. Respirology *23*, 220–227. https://doi.org/10.1111/resp.13179.
17. Raita, Y., Pérez-Losada, M., Freishtat, R.J., Harmon, B., Mansbach, J.M., Piedra, P.A., Zhu, Z., Camargo, C.A., and Hasegawa, K. (2021). Integrated omics endotyping of infants with respiratory syncytial virus bronchiolitis and risk of childhood asthma. Nat. Commun. *12*, 3601. https://doi.org/10.1038/s41467-021-23859-6.
18. Wildenbeest, J.G., Billard, M.N., Zuurbier, R.P., Korsten, K., Langedijk, A.C., van de Ven, P.M., Snape, M.D., Drysdale, S.B., Pollard, A.J., Robinson, H., et al. (2023). The burden of respiratory syncytial virus in healthy term-born infants in Europe: a prospective birth cohort study. Lancet Respir. Med. *11*, 341–353. https://doi.org/10.1016/S2213-2600(22)00414-3.
19. Johnson, M., Chelysheva, I., Öner, D., McGinley, J., Lin, G.L., O'Connor, D., Robinson, H., Drysdale, S.B., Gammin, E., Vernon, S., et al. (2024). A Genome-Wide Association Study of Respiratory Syncytial Virus Infection Severity in Infants. J. Infect. Dis. *229*, S112–S119. https://doi.org/10.1093/infdis/jiae029.
20. Jefferies, K., Drysdale, S.B., Robinson, H., Clutterbuck, E.A., Blackwell, L., McGinley, J., Lin, G.L., Galal, U., Nair, H., Aerssens, J., et al. (2020). Presumed Risk Factors and Biomarkers for Severe Respiratory Syncytial Virus Disease and Related Sequelae: Protocol for an Observational Multicenter, Case-Control Study From the Respiratory Syncytial Virus Consortium in Europe (RESCEU). J. Infect. Dis. *222*, S658–S665. https://doi.org/10.1093/infdis/jiaa239.
21. Wildenbeest, J.G., Zuurbier, R.P., Korsten, K., van Houten, M.A., Billard, M.N., Derksen-Lazet, N., Snape, M.D., Drysdale, S.B., Robinson, H., Pollard, A.J., et al. (2020). Respiratory Syncytial Virus Consortium in Europe (RESCEU) Birth Cohort Study: Defining the Burden of Infant Respiratory Syncytial Virus Disease in Europe. J. Infect. Dis. *222*, S606–S612. https://doi.org/10.1093/infdis/jiaa310.
22. Justicia-Grande, A.J., Pardo-Seco, J., Cebey-López, M., Vilanova-Trillo, L., Gómez-Carballa, A., Rivero-Calle, I., Puente-Puig, M., Curros-Novo, C., Gómez-Rial, J., Salas, A., et al. (2016). Development and Validation of a New Clinical Scale for Infants with Acute Respiratory Infection: The ReSVinet Scale. PLoS One *11*, e0157665. https://doi.org/10.1371/journal.pone.0157665.
23. Ederveen, T.H.A., Ferwerda, G., Ahout, I.M., Vissers, M., de Groot, R., Boekhorst, J., Timmerman, H.M., Huynen, M.A., van Hijum, S.A.F.T., and de Jonge, M.I. (2018). Haemophilus is overrepresented in the nasopharynx of infants hospitalized with RSV infection and associated with increased viral load and enhanced mucosal CXCL8 responses. Microbiome *6*, 10. https://doi.org/10.1186/s40168-017-0395-y.
24. Krohmaly, K.I., Perez-Losada, M., Ramos-Tapia, I., Zhu, Z., Hasegawa, K., Camargo, C.A., Jr., Harmon, B., Espinola, J.A., Reck Cechinel, L., Batabyal, R., et al. (2024). Bacterial small RNAs may mediate immune response differences seen in respiratory syncytial virus versus rhinovirus bronchiolitis. Front. Immunol. *15*, 1330991. https://doi.org/10.3389/fimmu.2024.1330991.
25. Lin, G.L., Drysdale, S.B., Snape, M.D., O'Connor, D., Brown, A., MacIntyre-Cockett, G., Mellado-Gomez, E., de Cesare, M., Ansari, M.A., Bonsall, D., et al. (2024). Targeted metagenomics reveals association between severity and pathogen co-detection in infants with respiratory syncytial virus. Nat. Commun. *15*, 2379. https://doi.org/10.1038/s41467-024-46648-3.
26. Thorburn, K., Harigopal, S., Reddy, V., Taylor, N., and van Saene, H.K.F. (2006). High incidence of pulmonary bacterial co-infection in children with severe respiratory syncytial virus (RSV) bronchiolitis. Thorax *61*, 611–615. https://doi.org/10.1136/thx.2005.048397.
27. Lin, H.C., Liu, Y.C., Hsing, T.Y., Chen, L.L., Liu, Y.C., Yen, T.Y., Lu, C.Y., Chang, L.Y., Chen, J.M., Lee, P.I., et al. (2022). RSV pneumonia with or without bacterial co-infection among healthy children. J. Formos. Med. Assoc. *121*, 687–693. https://doi.org/10.1016/j.jfma.2021.08.012.
28. Shein, S.L., Kong, M., McKee, B., O'Riordan, M., Toltzis, P., and Randolph, A.G. (2019). Antibiotic Prescription in Young Children With Respiratory Syncytial Virus–Associated Respiratory Failure and Associated Outcomes. Pediatr. Crit. Care Med. *20*, 101–109. https://doi.org/10.1097/PCC.0000000000001839.
29. de Koff, E.M., Man, W.H., Houten, MA van, Jansen, N.J.G., Arp, K., Hasrat, R., Sanders, E.A.M., and Bogaert, D. (2021). The respiratory microbiota during and following mechanical ventilation for respiratory infections in children. Eur. Respir. J. *57*, 2022652. https://doi.org/10.1183/13993003.02652-2020.
30. Man, W.H., Scheltema, N.M., Clerc, M., van Houten, M.A., Nibbelke, E.E., Achten, N.B., Arp, K., Sanders, E.A.M., Bont, L.J., and Bogaert, D. (2020). Infant respiratory syncytial virus prophylaxis and nasopharyngeal microbiota until 6 years of life: a subanalysis of the MAKI randomised controlled trial. Lancet Respir. Med. *8*, 1022–1031. https://doi.org/10.1016/S2213-2600(19)30470-9.
31. Brown, M.A., Jabeen, M., Bharj, G., and Hinks, T.S.C. (2022). Non-typeable Haemophilus influenzae airways infection: the next treatable trait in

asthma? Eur. Respir. Rev. *31*, 220008. https://doi.org/10.1183/16000617.0008-2022.

32. Gao, Y.D., Xepapadaki, P., Cui, Y.W., Stanic, B., Maurer, D.J., Bachert, C., Zhang, N., Finotto, S., Chalubinski, M., Lukkarinen, H., et al. (2023). Effect of Haemophilus influenzae, Streptococcus pneumoniae and influenza vaccinations on infections, immune response and asthma control in preschool children with asthma. Allergy *78*, 1473–1488. https://doi.org/10.1111/all.15551.
33. Teo, S.M., Tang, H.H.F., Mok, D., Judd, L.M., Watts, S.C., Pham, K., Holt, B.J., Kusel, M., Serralha, M., Troy, N., et al. (2018). Airway Microbiota Dynamics Uncover a Critical Window for Interplay of Pathogenic Bacteria and Allergy in Childhood Respiratory Disease. Cell Host Microbe *24*, 341–352.e5. https://doi.org/10.1016/j.chom.2018.08.005.
34. Andeweg, S.P., Schepp, R.M., van de Kassteele, J., Mollema, L., Berbers, G.A.M., and van Boven, M. (2021). Population-based serology reveals risk factors for RSV infection in children younger than 5 years. Sci. Rep. *11*, 8953. https://doi.org/10.1038/s41598-021-88524-w.
35. Biesbroek, G., Tsivtsivadze, E., Sanders, E.A.M., Montijn, R., Veenhoven, R.H., Keijser, B.J.F., and Bogaert, D. (2014). Early Respiratory Microbiota Composition Determines Bacterial Succession Patterns and Respiratory Health in Children. Am. J. Respir. Crit. Care Med. *190*, 1283–1292. https://doi.org/10.1164/rccm.201407-1240OC.
36. Galanti, M., Birger, R., Ud-Dean, M., Filip, I., Morita, H., Comito, D., Anthony, S., Freyer, G.A., Ibrahim, S., Lane, B., et al. (2019). Rates of asymptomatic respiratory virus infection across age groups. Epidemiol. Infect. *147*, e176. https://doi.org/10.1017/S0950268819000505.
37. Bogaert, D., Keijser, B., Huse, S., Rossen, J., Veenhoven, R., van Gils, E., Bruin, J., Montijn, R., Bonten, M., and Sanders, E. (2011). Variability and diversity of nasopharyngeal microbiota in children: a metagenomic analysis. PLoS One *6*, e17035. https://doi.org/10.1371/journal.pone.0017035.
38. Caporaso, J.G., Lauber, C.L., Walters, W.A., Berg-Lyons, D., Lozupone, C.A., Turnbaugh, P.J., Fierer, N., and Knight, R. (2011). Global patterns of 16S rRNA diversity at a depth of millions of sequences per sample. Proc. Natl. Acad. Sci. *108*, 4516–4522. https://doi.org/10.1073/pnas.1000080107.
39. Hasrat, R., Kool, J., de Steenhuijsen Piters, W.A.A., Chu, M.L.J.N., Kuiling, S., Groot, J.A., van Logchem, E.M., Fuentes, S., Franz, E., Bogaert, D., and Bosch, T. (2021). Benchmarking laboratory processes to characterise low-biomass respiratory microbiota. Sci. Rep. *11*, 17148. https://doi.org/10.1038/s41598-021-96556-5.
40. Bogaert, D., van Beveren, G.J., de Koff, E.M., Lusarreta Parga, P., Balcazar Lopez, C.E., Koppensteiner, L., Clerc, M., Hasrat, R., Arp, K., Chu, M.L.J.N., et al. (2023). Mother-to-infant microbiota transmission and infant microbiota development across multiple body sites. Cell Host Microbe *31*, 447–460.e6. https://doi.org/10.1016/j.chom.2023.01.018.
41. Callahan, B.J., McMurdie, P.J., Rosen, M.J., Han, A.W., Johnson, A.J.A., and Holmes, S.P. (2016). DADA2: High-resolution sample inference from Illumina amplicon data. Nat. Methods *13*, 581–583. https://doi.org/10.1038/nmeth.3869.
42. de Steenhuijsen Piters, W.A.A., Watson, R.L., de Koff, E.M., Hasrat, R., Arp, K., Chu, M.L.J.N., de Groot, P.C.M., van Houten, M.A., Sanders, E.A.M., and Bogaert, D. (2022). Early-life viral infections are associated with disadvantageous immune and microbiota profiles and recurrent respiratory infections. Nat. Microbiol 7, 224.
43. Edgar, R.C. (2018). Updating the 97% identity threshold for 16S ribosomal RNA OTUs. A. Valencia, ed. *34*, 2371–2375. https://doi.org/10.1093/bioinformatics/bty113.
44. Mölder, F., Jablonski, K.P., Letcher, B., Hall, M.B., Tomkins-Tinch, C.H., Sochat, V., Forster, J., Lee, S., Twardziok, S.O., Kanitz, A., et al. (2021). Sustainable data analysis with Snakemake. F1000Res. *10*, 33. https://doi.org/10.12688/f1000research.29032.1.
45. Mallick, H., Rahnavard, A., McIver, L.J., Ma, S., Zhang, Y., Nguyen, L.H., Tickle, T.L., Weingart, G., Ren, B., Schwager, E.H., et al. (2021). Multivariable association discovery in population-scale meta-omics studies. PLoS Comput. Biol. *17*, e1009442. https://doi.org/10.1371/journal.pcbi.1009442.

STAR★METHODS

KEY RESOURCES TABLE

REAGENT or RESOURCE	SOURCE	IDENTIFIER
Bacterial and virus strains		
ZymoBIOMICS microbial community standard	Zymo Research, CA, USA	Cat#D6300
ZymoBIOMICS microbial community DNA standard	Zymo Research, CA, USA	Cat#D6306
Biological samples		
Birth cohort study	Multiple sites	NCT03627572
Case-control study	Multiple sites	NCT03756766
COPAN eSwab, 482CE (transoral nasopharyngeal swab)	Copan Diagnostics Inc., CA, USA	Cat#482CE
Chemicals, peptides, and recombinant proteins		
Zirconium beads (0.1mm)	Biospec Products, OK, USA	Cat#11079101z
Phenol	VWR, PA, USA	Cat#A1153.0500
Master mix universal taqman 5 × 5 mL	Thermo Fischer Scientific, MA, USA	Cat#10556365
HPLC grade water	Instruchemie, The Netherlands	Cat#2195
10mM dNTP mix	Roche, Switzerland	Cat#11814362001
phiX control v3	Illumina, CA, USA	Cat#FC-110-3001
Critical commercial assays		
Mini-Beadbeater-24	Biospec Products, OK, USA	Cat#112011EUR
StepOnePlus Real-Time PCR System	Thermo Fisher Scientific, MA, USA	Cat#4376600
AlereTM i RSV	Abbott, Illinois, United States	Cat#435-000
Mag Mini DNA extraction kit	Immunosource, Belgium	Cat#NAP40401
Phusion Hot Start II High-Fidelity DNA Polymerase	Thermo Fisher Scientific, MA, USA	Cat#F-549L
Quant-iT PicoGreen dsDNA Assay Kit	Thermo Fisher Scientific, MA, USA	Cat#P7589
Beckman Coulter Agencourt AMPure XP	Thermo Fisher Scientific, MA, USA	Cat#A63880
MiSeq Reagent Kit v2 (2 × 250bp)	Illumina, CA, USA	Cat#MS-102-2003
Illumina MiSeq instrument	Illumina, CA, USA	Cat#SY-410-1003
Deposited data		
Raw 16S-rRNA sequencing data	This paper; NCBI SRA	Bioproject: PRJNA914884
Silva v138 (Version 2; August 2020)	(36)	https://zenodo.org/record/3986799#.YfD5ti-iH0r
Source code/scripts to process and analyze the data	This paper	https://doi.org/10.5281/zenodo.13941981
Oligonucleotides		
forward primer 16S-F1 5′-CGA AAG CGT GGG GAG CAA A-3′	(37)	N/A
reverse primer 16S-R1 5′-GTT CGT ACT CCC CAG GCG G-3′	(37)	N/A
probe 16S-P1 FAM-ATT AGA TAC CCT GGT AGT CCA-ZEN	(37)	N/A
515F 16S V4 forward primer 5′-GTGCCAGC MGCCGCGGTAA-3’ (including Illumina adapters and barcodes)	(38)	N/A
806R 16S V4 reverse primer 5′-GGACTACHVGGGTWTCTAAT-3’ (including Illumina adapters and barcodes)	(38)	N/A
Software and algorithms		
R Statistical Software v4.3.3	R Core Team	https://www.r-project.org

(Continued on next page)

Continued

REAGENT or RESOURCE	SOURCE	IDENTIFIER
RStudio v2024.04.1 + 748	RStudio	https://posit.co
Adobe Illustrator v26.3.1	Adobe	https://www.adobe.com/products/illustrator.html
python v3.8.13	N/A	https://www.python.org
DADA2 v1.16	(39)	https://benjjneb.github.io/dada2/
snakemake v5.18.1	(40)	https://snakemake.readthedocs.io/en/stable/
decontam v1.12.0	(41)	https://bioconductor.org/packages/release/bioc/html/decontam.html
lmerTest v3.1.3	N/A	https://cran.r-project.org/web/packages/lmerTest/index.html
phyloseq v1.41.1	(42)	https://joey711.github.io/phyloseq/
vegan v2.6-4	(43)	https://cran.r-project.org/web/packages/vegan/index.html
tidymodels v0.1.4	N/A	https://www.tidymodels.org
ranger v0.13.1	(44)	https://cran.r-project.org/web/packages/ranger/index.html
finetune v1.0.1	N/A	https://cran.r-project.org/web/packages/finetune/index.html
pROC v1.18.5	(39)	https://cran.r-project.org/web/packages/pROC/index.html
rsample v1.1.1	N/A	https://cran.r-project.org/web/packages/rsample/index.html
fastshap v0.1.1	(45)	https://cran.r-project.org/web/packages/fastshap/index.html

EXPERIMENTAL MODEL AND SUBJECT DETAILS

Study population and sample collection

Samples were collected in the context of two studies which were part of the REspiratory Syncytial virus Consortium in EUrope (RESCEU) project. Additional details on study design, recruitment and inclusion criteria of both studies were published previously.[20,21] In short, for both studies healthy, term born children were included. Children with significant cardiovascular, respiratory, renal, gastrointestinal, haematological, neurological, endocrine, immunological, musculoskeletal, oncological, or congenital disorders at time of inclusion were excluded from the study. Children who were diagnosed with comorbidity during the study were not excluded.

The first study is a birth cohort study, conducted in Spain, the United Kingdom, Finland and the Netherlands between 2017 and 2020 (ClinicalTrials.gov identifier: NCT03627572). Nasopharyngeal samples were collected during the first week of life. These infants were followed over the first year of life. During the RSV season, parents were instructed to contact the research nurse in the event of respiratory symptoms, and were called weekly to assess respiratory symptoms in the participating infants. During respiratory symptoms in the RSV season RSV diagnostics using PCR was performed. Part of the study sites (Spain, The Netherlands and United Kingdom) performed an RSV point-of-care test (Alere i RSV assay) at these visits, and collected samples for nasopharyngeal microbiome sequencing from RSV positive children. This design allowed us to also capture very mild RSV cases, who otherwise would not have sought medical attendance. We considered samples to be positive if RSV was detected by point of care test, PCR, or both.

The second study was a case-control study during which nasopharyngeal samples were collected from healthy term born infants younger than one year with RSV who were admitted in hospital, who visited the emergency department but were discharged within 12 h, or who visited the general practitioner (ClinicalTrials.gov identifier: NCT03756766). As a control group, nasopharyngeal samples were collected from age-matched healthy term born infants with no RSV infection. This control group was recruited both from the general population as well as from the hospital population, excluding children with a history of respiratory illness in the past 7 days, a history of concurrent clinically significant medical illness as judged by the investigator, premature birth, or vaccination in the past 7 days. In the RSV group, additional samples and questionnaires on residual complaints were collected six to eight weeks later, during the convalescent phase.

RSV was diagnosed using point-of-care qualitative molecular testing and/or by routine antigen or PCR tests at a central laboratory. A child was considered RSV positive if one or more tests were RSV positive.

Demographic and clinical data

Demographic and clinical data on medication use and disease severity was collected from all participants. Disease severity was measured using the ReSViNET score, which consists of a combination of clinical parameters.[22] In line with previous literature, we defined a mild RSV infection as a ReSViNET score of 0–7, a moderately severe infection as a ReSViNET score of 8–13, and severe infection as a ReSViNET score of 14–20.[22]

To further stratify severity within mild/moderate RSV cases, we investigated whether caretakers sought medical attendance or not (medically attended vs. not medically attended RSV infection). Full details on sample collection were previously published.[18,19] Briefly summarized, the sampling protocol for both studies was identical, collecting nasopharyngeal samples using nasal swabs by trained personnel. Samples were transported in Amies transport medium, and stored at -80°C until further workup.

This study was approved by the ethics committees at each study site and conducted in accordance with the European Statements for Good Clinical Practice. The birth cohort study was approved by the institutional review board of the University Medical Center Utrecht, the NHS National Research Ethics Service Oxfordshire Committee A, the South East Scotland Research Ethics Committee, the Ethics Committee of the Hospital District of Southwest Finland, and Hospital Clínico Universitario de Santiago de Compostela. The case-control study was approved by the NRES South Central and Hampshire A, the Comité de Ética de la Investigación de Santiago-Lugo, and the Medical Ethical Committee UMC Utrecht. Informed consent was obtained from the parents or legal guardians of all participants. Due to the young age of the participants (younger than one year of age) assent was not obtained.

METHOD DETAILS

Bacterial DNA isolation

Bacterial DNA was extracted using an Mag Mini DNA extraction kit (Immunosource, Belgium). Samples were thawed on ice and vortexed for 10 s. Per sample, 600μL of lysis buffer with zirconium beads (Biospec Products, OK, USA) and 550μL phenol (VWR, PA, USA) was added in a conical 1.5mL screw-cap Eppendorf tube. Samples were mechanically disrupted twice for 2 min at 3,500 oscillations/minute by bead beating and transferred on ice for 2 min after each bead-beating step (Mini-Beadbeater-24, Biospec Products, OK, USA). The tubes were centrifuged for 10 min at 4,500 × g. The clear aqueous phase was added to a 2mL Eppendorf tube containing 1.3mL binding buffer and 10μL magnetic beads. After shaking for 30 min, the tubes were placed in a magnetic separation rack. The supernatant was discarded, the magnetic beads were washed with wash buffer 1 and 2 and air-dried for 15 min at 55°C. DNA was eluted in 35μL, by shaking for 15 min at 55°C. Supernatant was transferred to a 1.5mL Eppendorf LoBind tube and stored at -20°C.

Negative (lysis buffer) and whole cell positive controls (Zymo Research, CA, USA) were included in DNA isolation runs.

Bacterial DNA quantification

Total bacterial density was assessed by quantitative PCR (StepOnePlus Real-Time PCR System, Thermo Fisher Scientific, MA, USA) with universal primers and probes targeting the 16S-rRNA gene.[37] The PCR mixture consisted of 12.5μL of 2 × master mix (Thermo Fisher Scientific, MA, USA), 1μL of each primer at 10 mM, 1μL of the probe at 5 mM, 6.5μL DNA free water and 3μL of template DNA. Amplifications were performed using a StepOnePlus Real-Time PCR System under the following conditions: 2 min at 50°C and 10 min at 95°C, followed by 45 cycles of 15 s at 95°C and 1 min at 60°C. C_T-values were related to a standard curve ranging from 0.1 pg/mL to 1.0 ng/mL of bacterial DNA (synthesized fragment of the 16S-rRNA gene).

16S-rRNA-sequencing

The bacterial DNA was amplified using barcoded primers (including the Illumina sequencing adapters), targeting the V4-region of the 16S-rRNA (515F/806R primers).[38] Each 25μL PCR reaction consisted of 0.5μL Phusion Hot Start II High-Fidelity DNA Polymerase (Thermo Fisher Scientific, MA, USA), 5μL 5×Phusion HF Buffer, 7μL HPLC grade water (Instruchemie, The Netherlands), 2.5μL of 2mM dNTP mix (Roche, Switzerland), 5μL of the combination barcoded 515F and 806R primers at 5μM and 5μL template DNA. PCR reactions were executed using the following cycling parameters; initial denaturation at 98°C for 30 s; 30 cycles of 10 s denaturation at 98°C, 30 s annealing at 55°C and 30 s elongation at 72°C, with a final extension at 72°C for 5 min. Samples with an input 16S-rRNA gene DNA concentration of <20 pg/μL (based on 16S-rRNA qPCR) were used undiluted, samples with a higher concentration were diluted in HPLC grade water, accordingly. DNA isolation blanks, no template controls (NTC; Milli-Q water), isolated whole cell and/or DNA positive controls (Zymo Research, CA, USA) were included in each PCR plate and amplified alongside the samples. The fragment size of the amplicon was assessed using agarose gel electrophoresis. Amplicons were subsequently quantified using Quant-iT PicoGreen dsDNA Assay, pooled at equimolar amounts and purified using 2× purification by 0.9×AMPure XP magnetic beads (Thermo Fisher Scientific, MA, USA). The library was prepared and spiked with phiX as recommended by Illumina and sequenced using the Miseq Reagent Kit v2 (paired-end; 2 × 250bp)[7] on an Illumina MiSeq instrument (Illumina, CA, USA). Details on bacterial DNA isolation and quantification and 16S-rRNA gene amplicon library preparation and sequencing have been previously published.[39,40]

Bioinformatic processing 16S-rRNA sequences

Using DADA2 (v1.16.0),[41] paired-end sequences were filtered and trimmed (maxEE = 2, truncLen = 200/150bp, truncQ = 2), denoised, merged (minOverlap = 12, maxMismatch = 0) and a sequence table was constructed. Chimeras were identified and

removed (method = 'consensus'). ASVs were annotated up to genus-level using the DADA2 implementation of the naive Bayesian classifier based on the Silva v138 (Version 2) reference database.[42] Species-level annotations were added using the addSpecies()-function.[43] DADA2 was implemented using the Snakemake workflow management system (v5.18.1).[44] ASVs not assigned to the kingdom Bacteria or annotated as Mitochondria (family) or Chloroplast (class) were removed.

Contamination detection and removal

Contaminating sequences were removed using the decontam R-package (v1.19.0) using the 'combined' method (default parameters). Manual checks were performed by inspection of bacterial density × frequency plots and DNA isolation run × frequency plots. Also, bacterial density and frequency were inspected according to the study site. All ASVs flagged as contaminants by decontam were manually inspected and subsequently removed. After decontamination, all samples with >5,000 reads were removed from the dataset (Figure S1A). In total 107 DNA isolation control samples and 20 MiSeq PCR controls were used to detect and remove contaminating sequences.

QUANTIFICATION AND STATISTICAL ANALYSES

All analyses were performed in R v4.3.3 within R studio v2024.04.1 + 748 (Boston, MA). All statistical tests were two-sided and *p*-values were corrected for multiple testing using Benjamini-Hochberg (BH) per comparison (referred to as *q*-values), unless otherwise specified. Exploratory data analyses were conducted to assess whether the data met the assumptions required for the statistical approach used. We did not formally test for assumptions. Statistical details of experiments can be found in Results and Figure legends, including information on the statistical tests used, number of individuals (*N*) or samples (*n*), and the definition of center and dispersion/precision measures. A summary of all models used, including information on the type of model, groups considered, dependent and independent variables can be found in Figure S4.

Microbiota data were normalized using total sum scaling into relative abundance.

Comparisons

Alpha- and beta-diversity analyses, cluster enrichment and differential abundance tests were primarily used to compare microbiota profiles between 1) healthy controls and samples collected during RSV/RSV convalescence and between RSV and RSV convalescence, 2) healthy controls and mild, moderate or severe RSV or between mild RSV and moderate/severe RSV infections, 3) convalescent samples compared 3a) across mild/moderate/severe RSV infections and 3b) between samples collected from children with compared to children without remaining symptoms and 4) baseline microbiota 4a) in relation to host- and environmental drivers and compared between children who will develop 4b) an RSV infection yes/no or 4c) a medically attended RSV infection yes/no. Comparisons 4b and 4c were additionally assessed using a random forest model including 39 ASVs and age at RSV infection as predictors.

Modeling approach

All multivariable linear or logistic mixed effects models (lmerTest::lmer() and lme4::glmer() R-functions, respectively) included age at the moment of sampling and gender (fixed effects) and study site (random effect). In models comparing RSV convalescent samples to samples collected during infection, we additionally adjusted for the time between RSV and RSV convalescence and included subject ID as random effect. Alpha-/beta-diversity and clustering analyses (see details below) were further used to describe early-life microbiota composition and to compare very early microbiota profiles (<5 days) and (stabilized) early profiles collected at $\geq$5 days). For comparisons 4b and 4c, we additionally accounted for birth season and presence of siblings (fixed effects) in our models, with age at RSV infection included in models where was assess the association between baseline microbiota profiles and medically attended RSV yes/no. The association between host- and environmental drivers (study site, season of birth, birth mode, feeding type, and presence of siblings) and the overall microbiota composition at baseline (comparison 4a) was assessed using PERMANOVA-tests (vegan::adonis2() R-function), while accounting for age (categories) and study site (restricted permutations). For a detailed overview of all included fixed, optional and random effects, see Figure S4.

Alpha-diversity analysis

Shannon diversity was primarily used as a measure for within-sample/alpha-diversity, leveraging both species richness and evenness (phyloseq::estimate_richness() R-function). In addition, we compared the number of observed ASVs between healthy controls, RSV and convalescent samples to verify our Shannon-based conclusions (phyloseq::estimate_richness() R-function). The unfiltered and non-rarefied ASV table was used to calculate alpha- diversity. All alpha-diversity analyses were additionally adjusted for scaled sequencing depth (lmerTest R-package).

Beta-diversity analysis

For beta-diversity analyses, we filtered the data, only including ASVs present at >0.1% relative abundance in $\geq$2 samples. Bray-Curtis dissimilarity (vegan::vegdist() R-function) was used to determine the compositional dissimilarity between samples. To test for significant differences in the overall microbial community composition between groups we used univariable PERMANOVA

(Permutational Multivariate Analysis of Variance; vegan::adonis2() R-function). We additionally explored stability (Bray-Curtis dissimilarity) between paired samples collected during RSV infection and at convalescence, stratified by disease severity (mild, moderate or severe). Linear mixed effects models were used to assess statistical significance of the association with RSV severity with the Bray-Curtis dissimilarity between sample pairs (stability) as outcome (lmerTest R-package).

Clustering

Complete linkage hierarchical clustering (stats::hclust() R-function) based on the Bray-Curtis dissimilarity matrix was performed to generate cluster allocations. Clustering was performed once across the total dataset. The optimal number of clusters was determined using the Silhouette- and Calinski-Harabasz-indices (fpc R-package). In contrast to previous work on early-life nasopharyngeal microbiota,[7] we found characteristics of the well-known *Corynebacterium* and *Dolosigranulum* (CDG)-cluster spread across two clusters (CDGa and CDGb). Given this cluster is generally associated with a healthy phenotype, we decided to combine these clusters into one cluster (CDG), to be able to use this cluster as the ‘baseline’ to which other clusters were compared. A sensitivity analysis was performed to test to what degree clustering was affected by including multiple samples of the same subject. Therefore, we created a reduced dataset including a single sample from each subject by including 1) all baseline samples (birth cohort study), 2) all healthy control samples (case-control study), 3) all samples from individuals from whom we only had a single sample available (RSV/RSV-convalesent samples; case-control study) and 4) one random sample from individuals from whom we had both an RSV and RSV convalescent sample available (case-control study) (n = 1,111 samples). Clustering was repeated as before and cluster membership was compared for all samples included in the reduced dataset. Statistical significance of associations between cluster membership and outcomes (e.g., healthy vs. RSV) was determined using logistic mixed effects regression analyses (lme4 R-package).

Differential abundance analysis

Differential abundant ASVs between groups were identified using linear mixed effects models, similar to the implementation in the MaAsLin2-framework (default parameters; $\log_2$-normalisation; lmerTest R-package).[45] For each comparison ASVs present at >0.1% relative abundance in $\geq$5% of samples were selected. q-Values of below 0.05 were considered statistically significant.

Random forest modeling

To validate the results from our cluster-based logistic mixed effects regression models and MaAsLin2-analyses, classification random forest models based on baseline/premorbid microbiota were used to develop a classifier to determine if a child would go on to develop a medically attended or not medically attended RSV infection. Given the limited number of samples available, we filtered our data to include ASVs present in >5% of samples at $\geq$0.1% relative abundance, additionally including age at RSV infection. Modeling was performed within the tidymodels-framework, using ranger implementation of random forest models. Minimal node size [min_n] and number of trees [trees]-parameters were tuned. Tuning was performed using 5-fold cross-validation over 5 repeats combined with the finetune R-package (tune_race_anova()-function, automatically testing 25 candidate parameter sets). The model with the highest area-under-the-curve (AUC) receiver operating curve (ROC) was selected. Subsequently, we 1) fitted a final model using optimized parameters, used to explore out-of-bag estimates and feature importance and 2) used the optimized parameters in another round of 5-fold cross-validation, generating cross-validated predictions. AUC-ROCs were built using the pROC R-package. 95% confidence intervals for AUCs were calculated using the pROC::ci()-function (Delong method). To test the significance of the AUCs found, we performed a permutation test over 1,000 iterations (rsample R-package). Feature importance was assessed using permutation-based Shapley Additive Explanations (SHAP)-values (fastshap R-package). Using the same methodology, we fitted a model (without age at first RSV infection as predictor) to discriminate infants who get infected with RSV within the first year of life from those who do not.

Cell Reports Medicine, Volume *5*

Supplemental information

The respiratory microbiome is linked to the severity of RSV infections and the persistence of symptoms in children

Maartje Kristensen, Wouter A.A. de Steenhuijsen Piters, Joanne Wildenbeest, Marlies A. van Houten, Roy P. Zuurbier, Raiza Hasrat, Kayleigh Arp, Mei Ling J.N. Chu, Marie Billard, Terho Heikkinen, Steve Cunningham, Matthew Snape, Simon B. Drysdale, Ryan S. Thwaites, Federico Martinon-Torres, Andrew J. Pollard, Peter J.M. Openshaw, Jeroen Aerssen, Justyna Binkowska, Louis Bont, Debby Bogaert, and REspiratory Syncytial virus Consortium in EUrope (RESCEU) investigators

Cell Reports Medicine, Volume *5*

Supplemental information

The respiratory microbiome is linked to the severity of RSV infections and the persistence of symptoms in children

Maartje Kristensen, Wouter A.A. de Steenhuijsen Piters, Joanne Wildenbeest, Marlies A. van Houten, Roy P. Zuurbier, Raiza Hasrat, Kayleigh Arp, Mei Ling J.N. Chu, Marie Billard, Terho Heikkinen, Steve Cunningham, Matthew Snape, Simon B. Drysdale, Ryan S. Thwaites, Federico Martinon-Torres, Andrew J. Pollard, Peter J.M. Openshaw, Jeroen Aerssen, Justyna Binkowska, Louis Bont, Debby Bogaert, and REspiratory Syncytial virus Consortium in EUrope (RESCEU) investigators

Supplemental information

Variable	Overall, N = 773[1]	No RSV infection in the first year of life, N = 576[1]	RSV infection in the first year of life, N = 197[1]	p-value[2]
Year of birth				0.004[a]
2017	56/773 (7.2%)	41/576 (7.1%)	15/197 (7.6%)	
2018	336/773 (43%)	270/576 (47%)	66/197 (34%)	
2019	381/773 (49%)	265/576 (46%)	116/197 (59%)	
Season of birth				0.459[a]
Autumn	186/773 (24%)	146/576 (25%)	40/197 (20%)	
Spring	198/773 (26%)	148/576 (26%)	50/197 (25%)	
Summer	225/773 (29%)	165/576 (29%)	60/197 (30%)	
Winter	164/773 (21%)	117/576 (20%)	47/197 (24%)	
Gestational age (weeks)				0.942[a]
37+0-37+6	67/760 (8.8%)	52/568 (9.2%)	15/192 (7.8%)	
38+0-38+6	98/760 (13%)	74/568 (13%)	24/192 (12%)	
39+0-39+6	240/760 (32%)	178/568 (31%)	62/192 (32%)	
>=40+0	355/760 (47%)	264/568 (46%)	91/192 (47%)	
Vaginal delivery	212/773 (27%)	156/576 (27%)	56/197 (28%)	0.715[a]
Birth weigth	3,490 (3,158, 3,780)	3,470 (3,122, 3,789)	3,516 (3,286, 3,748)	0.084[b]
Female sex	380/773 (49%)	290/576 (50%)	90/197 (46%)	0.259[a]
Apgar score (1 minute)	9.00 (9.00, 9.00)	9.00 (9.00, 9.00)	9.00 (9.00, 9.00)	0.147[b]
Apgar score (5 minutes)	10.00 (9.00, 10.00)	10.00 (9.00, 10.00)	10.00 (9.00, 10.00)	0.872[b]
Apgar score (10 minutes)	10.00 (9.00, 10.00)	10.00 (9.00, 10.00)	10.00 (9.00, 10.00)	0.310[b]
Postnatal infection	18/770 (2.3%)	15/574 (2.6%)	3/196 (1.5%)	0.584[c]
Postnatal antibiotic treatment	13/18 (72%)	12/15 (80%)	1/3 (33%)	0.172[c]
Asphyxia	3/769 (0.4%)	2/573 (0.3%)	1/196 (0.5%)	>0.999[c]
Hospitalisation	78/773 (10%)	54/576 (9.4%)	24/197 (12%)	0.259[a]
Hospital length of stay	2.00 (2.00, 3.00)	2.00 (2.00, 3.00)	2.00 (2.00, 3.00)	0.699[b]
Any respiratory support	8/766 (1.0%)	5/571 (0.9%)	3/195 (1.5%)	0.427[c]
Mother planning to breastfeed	680/771 (88%)	506/574 (88%)	174/197 (88%)	0.949[a]
Intention to register to daycare	368/764 (48%)	259/568 (46%)	109/196 (56%)	0.016[a]
Maternal smoking during pregnancy	40/771 (5.2%)	34/574 (5.9%)	6/197 (3.0%)	0.116[a]
Household smoking	101/770 (13%)	81/573 (14%)	20/197 (10%)	0.153[a]
Pets inside the home	319/771 (41%)	253/574 (44%)	66/197 (34%)	0.009[a]
Any family member with history of asthma	206/768 (27%)	146/572 (26%)	60/196 (31%)	0.165[a]
Number of RTI episodes	1.00 (0.00, 3.00)	1.00 (0.00, 2.00)	3.00 (2.00, 4.00)	<0.001[b]
Age at first RSV infection	170 (108, 260)	N/A	170 (108, 260)	
Medically attended RSV infection	100/185 (54%)	N/A	100/185 (54%)	
Severity of first RSV infection				
mild	173/190 (91%)	N/A	173/190 (91%)	
moderate	17/190 (8.9%)	N/A	17/190 (8.9%)	

Table S1. Baseline characteristics of early-life samples, stratified by whether or not the child would develop an RSV infection in the first year of life.

[1]n/N (%); Median (IQR)

[2]To assess statistical significance between groups (subjects who will develop an RSV infection over the first year of life and those who do not) we used a [a]Pearson's Chi-squared test; [b]Wilcoxon rank sum test or a [c]Fisher's exact test.

Variable	Healthy controls, N = 52[1]	RSV, N = 374[1]	p-value[2]
Study			<0.001[a]
Birth cohort	0/52 (0%)	117/374 (31%)	
Case-control	52/52 (100%)	257/374 (69%)	
Age (days)	222 (112, 297)	94 (48, 200)	<0.001[b]
Female sex	15/52 (29%)	174/373 (47%)	0.016[a]
Year (visit)			<0.001[c]
2017	0/52 (0%)	22/254 (8.7%)	
2018	11/52 (21%)	106/254 (42%)	
2019	41/52 (79%)	100/254 (39%)	
2020	0/52 (0%)	26/254 (10%)	
Season (visit)			<0.001[c]
Autumn	21/52 (40%)	163/254 (64%)	
Spring	11/52 (21%)	1/254 (0.4%)	
Summer	19/52 (37%)	0/254 (0%)	
Winter	1/52 (1.9%)	90/254 (35%)	
Site			0.063[c]
Spain	6/52 (12%)	27/347 (7.8%)	
The Netherlands	25/52 (48%)	181/347 (52%)	
UK (London)	3/52 (5.8%)	3/347 (0.9%)	
UK (Oxford)	18/52 (35%)	136/347 (39%)	
Duration onset of symptoms to sampling (days)	N/A	4.00 (3.00, 5.00)	
ReSViNET score	N/A	6.0 (4.0, 10.0)	
Fever (>38.5°C)	0/52 (0%)	317/374 (85%)	<0.001[a]
Any medical attendance	0/52 (0%)	301/370 (81%)	<0.001[a]
Hospitalisation	0/52 (0%)	192/367 (52%)	<0.001[a]
PICU admission	0/52 (0%)	61/241 (25%)	<0.001[a]
Any comorbidities	29/51 (57%)	40/257 (16%)	<0.001[a]

Table S2. Baseline characteristics of healthy controls compared to RSV cases.

[1]n/N (%); Median (IQR)

[2]To assess statistical significance between groups (healthy controls compared to RSV cases) we used a [a]Pearson's Chi-squared test; [b]Wilcoxon rank sum test or a [c]Fisher's exact test.

Variable	Mild RSV, N = 218[1]	Moderate RSV, N = 106[1]	Severe RSV, N = 47[1]	p-value[2]
Study				<0.001[a]
Birth cohort	106/218 (49%)	10/106 (9.4%)	1/47 (2.1%)	
Case-control	112/218 (51%)	96/106 (91%)	46/47 (98%)	
Age (days)	158 (62, 247)	87 (48, 184)	48 (28, 96)	<0.001[b]
Female sex	106/217 (49%)	49/106 (46%)	18/47 (38%)	0.418[a]
Year (visit)				0.738[c]
2017	8/112 (7.1%)	9/95 (9.5%)	5/44 (11%)	
2018	45/112 (40%)	40/95 (42%)	20/44 (45%)	
2019	50/112 (45%)	34/95 (36%)	15/44 (34%)	
2020	9/112 (8.0%)	12/95 (13%)	4/44 (9.1%)	
Season (visit)				0.031[c]
Autumn	82/112 (73%)	55/95 (58%)	24/44 (55%)	
Spring	0/112 (0%)	1/95 (1.1%)	0/44 (0%)	
Winter	30/112 (27%)	39/95 (41%)	20/44 (45%)	
Site				0.007[c]
Spain	9/194 (4.6%)	15/104 (14%)	2/46 (4.3%)	
The Netherlands	112/194 (58%)	42/104 (40%)	27/46 (59%)	
UK (London)	2/194 (1.0%)	0/104 (0%)	1/46 (2.2%)	
UK (Oxford)	71/194 (37%)	47/104 (45%)	16/46 (35%)	
Duration onset of symptoms to sampling (days)	3.00 (3.00, 5.00)	4.00 (3.00, 5.00)	4.00 (3.00, 5.00)	0.072[b]
ReSViNET score	4.0 (3.0, 6.0)	10.0 (8.2, 11.8)	16.0 (14.5, 17.0)	<0.001[b]
Fever (>38.5°C)	200/218 (92%)	80/106 (75%)	36/47 (77%)	<0.001[a]
Any medical attendance	152/214 (71%)	101/106 (95%)	47/47 (100%)	<0.001[a]
Hospitalisation	53/214 (25%)	93/105 (89%)	45/45 (100%)	<0.001[a]
PICU admission	4/101 (4.0%)	23/93 (25%)	34/46 (74%)	<0.001[a]
Any comorbidities	8/112 (7.1%)	19/96 (20%)	13/46 (28%)	0.002[a]

Table S3. Baseline characteristics of RSV cases, stratified by severity.

[1]n/N (%); Median (IQR)

[2]To assess statistical significance between groups (mild, moderate and severe RSV) we used a [a]Pearson's Chi-squared test; [b]Kruskal-Wallis rank sum test or a [c]Fisher's exact test.

Study	Group	Mean	SD	Min	p25	median	p75	Max
Birth cohort	Baseline	31,141	15,553	5,008	19,579	30,777	41,453	76,940
	RSV	35,680	7,913	12,298	30,640	36,063	39,904	63,236
	RSV (conv)	37,500	7,948	18,534	32,579	36,136	40,296	63,245
Case-control	Healthy controls	27,218	5,120	12,091	24,793	26,542	30,150	40,964
	RSV	27,207	8,100	5,383	22,060	26,952	30,957	57,902
	RSV (conv)	27,509	7,150	13,426	22,106	26,621	31,917	46,772
Overall	Overall	30,586	12,620	5,008	22,580	29,890	37,878	76,940

Table S4. Sequencing depth stratified by study and study group. Number of reads (after quality control/decontamination) stratified by study and study groups. 'Overall' represents the sequencing depth across the total dataset. Mean, standard deviation (SD), minimum (Min; p0), maximum (Max; p100), Median (p50) and 25th (p25) and 75th (p75) percentiles are shown.

Figure S1

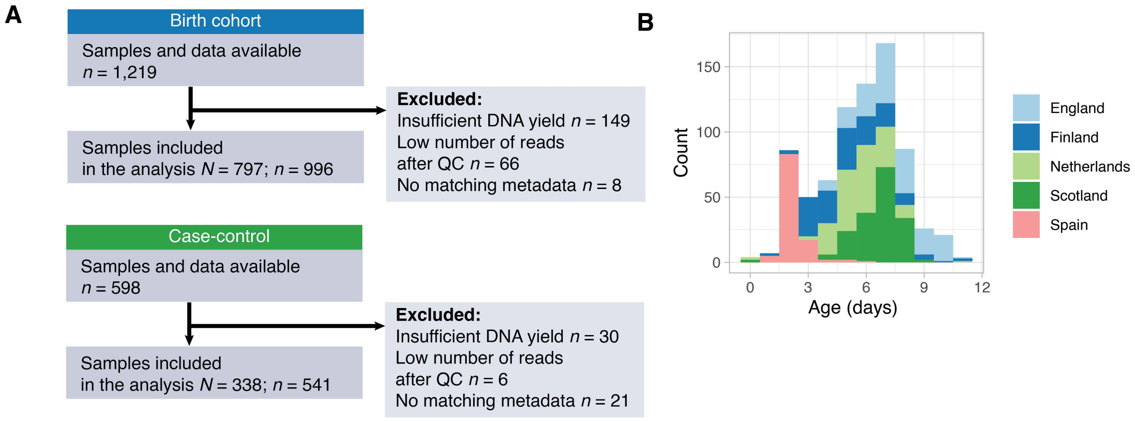

Figure S1. Sample inclusion and age at baseline sample collection. (A) Flow chart indicating the number of participants (N) and samples (n) at each step during laboratory processing and data preparation. (B) Histogram showing the age at sample collection of baseline samples per study site.

Figure S2

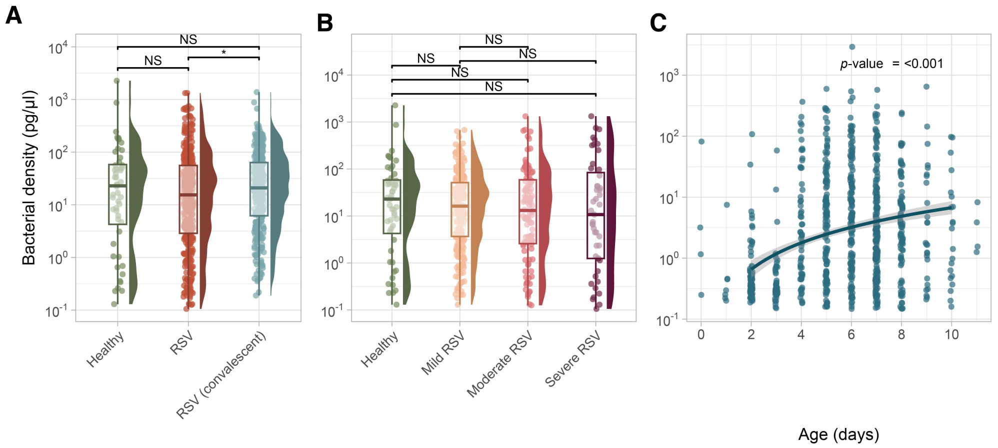

Figure S2. Bacterial density between groups and across age. (A) Bacterial density in healthy controls, during and after RSV infection and (B) in healthy controls (*n* = 52), mild (RESViNet-score 0-7; *n* = 218), moderate (8-13; *n* = 106) and severe RSV infection (14-20; *n* = 47). Statistical significance was assessed using linear mixed effects models with $\log_{10}$-transformed bacterial density as outcome, age, sex and health status (healthy controls, RSV or RSV convalescent; A) or RSV severity (healthy, mild, moderate and severe RSV; B) as fixed effects and study site as random effect. Subject ID was additionally included for comparisons between RSV and RSV convalescence. Box plots represent the 25th and 75th percentiles (lower and upper boundaries of boxes, respectively), the median (middle horizontal line), and measurements that fall within 1.5 times the interquartile range (IQR; distance between 25th and 75th percentiles; whiskers). (C) Bacterial density for baseline samples over age. Statistical significance was assessed using a linear mixed effects model, including $\log_{10}$-transformed bacterial density as outcome, the natural log of age, season of birth, sex and presence of siblings as fixed effects and study site as random effect. Asterisks denote statistical significance (NS, not significant [$p > 0.05$]; *, $p \leq 0.05$; **, $p \leq 0.01$; ***, $p \leq 0.001$).

Figure S3

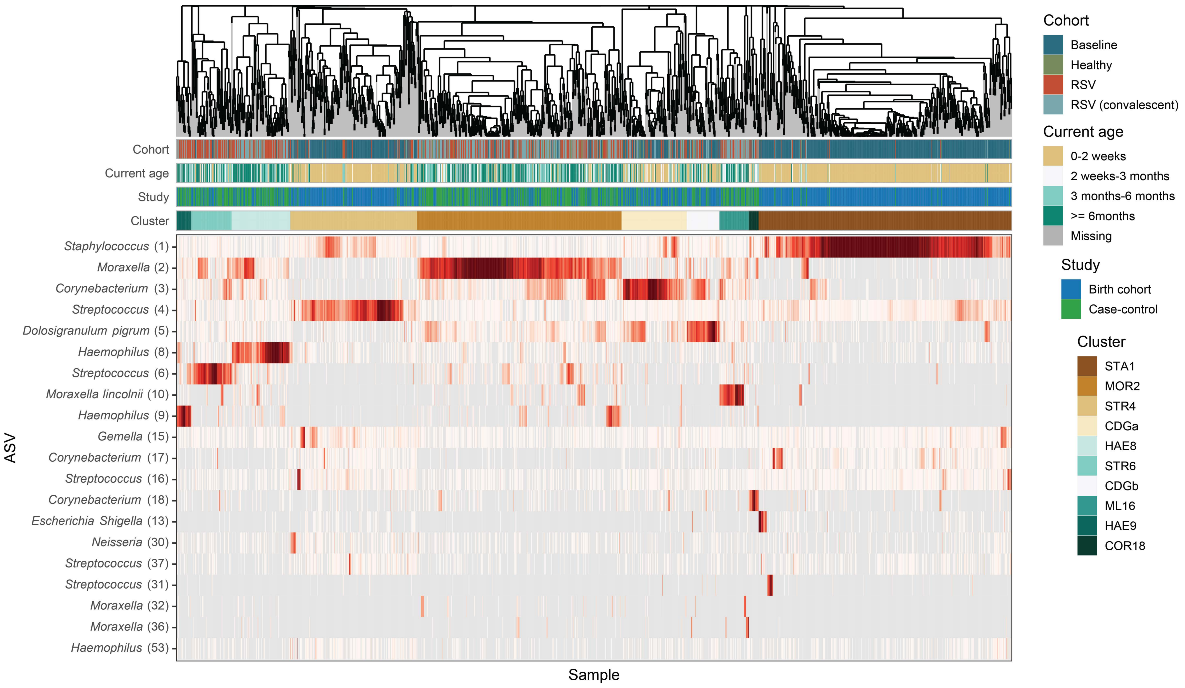

Figure S3. Dendrogram visualizing a complete linkage hierarchical clustering of samples based on the Bray-Curtis dissimilarity matrix. The length of the branches of the tree structure corresponds with the similarities between samples (*n* = 1,537). Adjacent to the branch ends information on 1) cohort [baseline, healthy controls, RSV and RSV convalescent], 2) current age, 3) study [birth cohort or case-control] and 4) cluster membership is depicted. Based on Calinski- Harabasz and Silhouette-indices we found an optimum of 10 clusters. These included clusters characterized by *Staphylococcus* (1) (STA1; *n* = 466), *Moraxella* (2) (MOR2; *n* = 376), *Streptococcus* (4) (STR4; *n* = 233), *Corynebacterium* (3)/*Dolosigranulum pigrum* (5) (CDGa and CDGb; *n* = 180), *Haemophilus* (8) (HAE8; *n* = 108), *Streptococcus* (6) (STR6; *n* = 74), *Moraxella lincolnii* (16) (ML16; *n* = 54), *Haemophilus* (9) (HAE9; *n* = 28) or *Corynebacterium* (18) (COR18; *n* = 18). A heatmap shows the relative abundance of the 20 highest-ranked ASVs based on mean relative abundance across all samples. Repeated samples from individuals were included in this clustering analysis to optimize cluster identification and increase comparability across time points.

Figure S4

Group(s)	Model	Dependent variable	Independent variables	Figure
HC RSVc / RSV HC	lmer	α-diversity	health status, age, sex, read count*, study site	2A
RSV RSVc	lmer	α-diversity	health status, age, sex, read count*, time RSV/RSVc, study site, subject ID	2A
HC RSVc / RSV HC	lmer	density†	health status, age, sex, study site	S2A
RSV RSVc	lmer	density†	health status, age, sex, study site, subject ID	S2A
HC RSVc / RSV HC	PERMANOVA	β-diversity	health status, age, sex, study site	2B
RSV RSVc	PERMANOVA	β-diversity	health status, age, sex, time RSV/RSVc, study site	
RSV RSVc	PERMANOVA	β-diversity	health status, age, subject ID	
HC RSVc / RSV HC	lmer	health status	cluster, age, sex, study site	2D
HC RSVc / RSV HC	MaAsLin2	ASV/genus†	health status, age, sex, study site	2E-F
HC mild / HC mod / HC sev	lmer	α-diversity	severity, age, sex, read count*, study site	3A
HC mild / HC mod / HC sev	lmer	density†	severity, age, sex, study site	S2B
HC mild / HC mod / HC sev	PERMANOVA	β-diversity	severity, age, sex, study site	3B
RSVc mild / RSVc mod / RSVc sev	lmer	stability	severity, age, sex, time RSV/RSVc, study site	3C
HC mild / HC mod / HC sev	lmer	severity	cluster, age, sex, study site	3D
HC mild / HC mod / HC sev	MaAsLin2	ASV/genus†	severity, age, sex, study site	3E-F
HC mild / HC mod / HC sev	MaAsLin2	ASV/genus†	severity, age, sex, time RSV/RSVc, abx, study site (within RSVc)	
<symptoms>	MaAsLin2	ASV/genus†	symptom, age, sex, time RSV/RSVc, study site (within RSVc)	4A-B
baseline	lmer	α-diversity	age†, sex, read count*, birth season, siblings, study site	5A
baseline	lmer	density†	age†, sex, birth season, siblings, study site	S2C
baseline	PERMANOVA	β-diversity	age (cat), study site (+/- Spain)	5B
baseline	PERMANOVA	β-diversity	study site, age (cat)	
baseline	PERMANOVA	β-diversity	birth season, age (cat), study site	
baseline	PERMANOVA	β-diversity	birth mode, age (cat), study site	
baseline	PERMANOVA	β-diversity	feeding, age (cat), study site	
baseline	PERMANOVA	β-diversity	sex, age (cat), study site	
baseline	PERMANOVA	β-diversity	siblings, age (cat), study site	
no RSV any RSV	lmer	RSV y/n	cluster, age, sex, birth season, siblings, study site	
no RSV any RSV	PERMANOVA	β-diversity	RSV y/n, age, sex, birth season, siblings, study site	
non-MA RSV MA RSV	lmer	MA RSV y/n	cluster, age, sex, age RSV, birth season, siblings, study site	5D
non-MA RSV MA RSV	PERMANOVA	β-diversity	MA RSV y/n, age, sex, age RSV, birth season, siblings, study site	
no RSV any RSV	rf	RSV y/n	ASV1, ASV2 ... ASV39, age RSV	
non-MA RSV MA RSV	rf	MA RSV y/n	ASV1, ASV2 ... ASV39, age RSV	5E-G

Variable of interest · Fixed effects · Optional effect · Random effects/blocked permutations

Figure S4. Overview of statistical models. Overview of all (multivariable) models included in either main text, (supplementary) figures or both. For each model, the type of model, dependent variables and independent variables (stratified by fixed effects or random effects) are indicated. Models were ran with and without 'optional effects'. Linear or logistic mixed effects models (lmerTest::lmer or lme4::glmer, respectively) were applied for continuous or dichotomous dependent variables, respectively, including study site and/or subject ID as random effect throughout. Blocked permutations were applied in PERMANOVA-tests to account for study site and/or subject ID. * scaled; † $\log_{10}$- or natural log-transformed; HC, healthy controls; RSVc, RSV convalescence; mod, moderate RSV infection; sev, severe RSV infection; age (cat), age categories; MA, medically attended; rf, random forest.
